# Community demand for comprehensive primary health care from malaria volunteers in South-East Myanmar: a qualitative study

**DOI:** 10.1186/s12936-020-03555-4

**Published:** 2021-01-06

**Authors:** Win Han Oo, Elizabeth Hoban, Lisa Gold, Kyu Kyu Than, Thazin La, Aung Thi, Freya J. I. Fowkes

**Affiliations:** 1 School of Health and Social Development, Faculty of Health, Deakin University, Melbourne, VIC Australia; 2 Disease Elimination Programme, Burnet Institute, Melbourne, VIC Australia; 3 Melbourne School of Population and Global Health, University of Melbourne, Melbourne, VIC Australia; 4 Department of Public Health, Myanmar Ministry of Health and Sports, Nay Pyi Taw, Myanmar; 5 Department of Epidemiology and Preventive Medicine, Monash University, Melbourne, VIC Australia

**Keywords:** Volunteer, Community-delivered model, Malaria elimination, Primary health care, Myanmar

## Abstract

**Background:**

Malaria volunteers have contributed significantly to malaria control achieving a reduction of annual parasite incidence to pre-elimination levels in several townships across Myanmar. However, the volunteers’ role is changing as Myanmar transitions from a malaria control to elimination programme and towards the goal of universal health coverage. The aim of the study is to explore the perspectives of community leaders, members and malaria volunteers in South-East Myanmar on community-delivered models to inform an optimal design that targets malaria elimination in the context of primary health care in Myanmar.

**Methods:**

Qualitative methods including focus group discussions (FGDs) with community members and current or ex-malaria volunteers, and participatory workshops with community leaders were conducted. All data collection tools were pilot tested with similar participants. The FGDs were stratified into male and female participants in consideration of diverse gender roles among the ethnic groups of Myanmar. Data saturation was the key cut-off point to cease recruitment of participants. Inductive thematic analysis was used.

**Results:**

Community members were willing to be tested for malaria because they were concerned about the consequences of malaria although they were aware that malaria prevalence is low in their villages. Malaria volunteers were the main service providers for malaria and other infectious diseases in the community. Apart from malaria, the community identified common health problems such as the flu (fever, sneezing and coughing), diarrhoea, skin infections and tuberculosis as priority diseases in this order. Incorporating preventive, and whenever possible curative, services for those diseases into the current malaria volunteer model was recommended.

**Discussion and conclusion:**

There was a gap between the communities’ expectations of health services and the health services currently being delivered by volunteers in the community that highlights the need for reassessment and reform of the volunteer model in the changing context. An evidence-based, community preferred, pragmatic community-delivered integrated model should be constructed based on the context of malaria elimination and progressing towards universal health coverage in Myanmar.

## Background

Malaria is a disease that disproportionately affects populations in hard-to-reach areas. Several malaria implementation models, in particular community-delivered models, have contributed to the decline in the malaria burden worldwide [1]. Community-delivered models increase coverage of bed net usage and intermittent preventive treatment in pregnancy, and improve malaria-metric outcomes, such as malaria mortality compared to non-community-delivered models [2]. The use of community-delivered models for malaria control is increasing because they are effective and economical [3–5].

Importantly, the success of malaria implementation models relies significantly on community participation and accountability to the affected populations. Many malaria implementation models have been successful because of the integration of community voices in their design, implementation and evaluation, but some programmes have failed because of inadequate or minimal consideration of community voices [4, 6].

Myanmar is a malaria endemic country in the Greater Mekong Sub-region (GMS) and it aims to eliminate malaria by 2030 [7, 8]. Volunteers were first used in Myanmar for malaria control in 2004, when the Myanmar Council of Churches commenced a community-delivered malaria control project focusing on early diagnosis and treatment in 160 remote villages in eight townships [9]. Other malaria implementing partners, including the Myanmar National Malaria Control Programme, recognized the usefulness of malaria volunteers and applied the model throughout the country. Currently, over 15,000 out of 67,285 villages in Myanmar (approximately 23%) have at least one malaria volunteer [10]. Myanmar has successfully used the malaria volunteer model, in conjunction with the government health facility-based model, to reduce the annual parasite incidence to below 1 in several townships within recent decades.

Community-based malaria control is effective and economical in various settings in Myanmar [11]. However, due to the reduction in malaria prevalence in Myanmar the focus has shifted to elimination and, therefore, the model needs to be reassessed and adapted to fit into Myanmar’s malaria elimination programme. The 1-3-7 strategy was adopted in Myanmar’s elimination programme that includes: case notification to National Malaria Control Programme within one day; case investigation at community level within three days; foci investigation and response, identification of additional fever cases and appropriate interventions within seven days after diagnosis of a malaria case [12]. It is questionable whether the current community-delivered malaria volunteer model is ready to host the 1-3-7 strategy without any adaptation because the existing community-delivered malaria models are designed only for malaria control and not for near real time notification of malaria cases within 24 h after diagnosis and volunteers assisting in case and foci investigations.

Paralleling the transition from malaria control to elimination, the motivation and social role of malaria volunteers is plummeting in tandem with the decline in the prevalence of malaria in Myanmar [13]. Evidence shows that in the context of lower malaria incidence, increasing the services that the community request, and the malaria volunteers can provide, increases utilization of volunteer’s services and enables more malaria testing in the community [14, 15]. Therefore, to maintain the Myanmar community-delivered model’s effectiveness and popularity, Myanmar National Malaria Control Programme rolled out the Integrated Community Malaria Volunteer (ICMV) programme throughout Myanmar in 2017–18. The ICMVs provide services for malaria, dengue, lymphatic filariasis, tuberculosis (TB), human immunodeficiency virus/acquired immunodeficiency syndrome (HIV/AIDS) and leprosy in areas that lack formal health care providers [16] (Additional file 1). Nevertheless, the services selected for inclusion in this package were not a result of community consultations and an evidence-based approach, but according to the political landscape of the Myanmar Ministry of Health and Sports and operational feasibility.

An evidence-based community-delivered malaria elimination model that integrates community perspectives in its design is required to ensure that the national malaria elimination programme is fit-for-purpose in the Myanmar primary health care sector. In order to bring community voices to the front and integrate their perspectives in the development of a community-delivered malaria elimination model for Myanmar, this paper provides the findings from a qualitative study conducted in Kayah and Kayin States in Myanmar in 2018.

## Methods

The study employed a qualitative research approach. Eight focus group discussions (FGDs) and two participatory workshops were conducted using guides. The researchers obtained community perspectives from community members (also known as villagers) or malaria volunteers (n = 72), and community leaders (also known as village leaders) (n = 18) (Table 1). During data collection, malaria volunteers did not officially identify themselves as such, choosing instead to identify as villagers. However, they provided volunteer perspectives in the FGDs. The participants were gender balanced in FGDs, but contributions by men dominated the participatory workshops. All participants were adults (aged over 18 years). About half (n = 44) of the participants were aged 31 to 45 years.

**Table 1:** Gender profile of the focus group discussion and participatory workshop participants

No	Township	Male	Female	Total no. of participants
FGDs with community members and malaria volunteers				
1	Demoso	5	5	10
2	Demoso	7	3	10
3	Demoso	6	4	10
4	Demoso	5	5	10
5	Leik Tho	4	4	8
6	Leik Tho	2	6	8
7	Leik Tho	2	6	8
8	Leik Tho	5	3	8
		36	36	72
Participatory workshops with community leaders				
1	Demoso	5	5	10
2	Taunggo	8	0	8
		13	5	18

The study ensured representation of diverse subnational groups in South East Myanmar reflecting different opinions of community groups [17, 18]. Myanmar is home to 135 ethnic groups who speak their own dialects and possess unique customs. To engage these populations in the study, FGDs were held in two ethnically diverse states in South-East Myanmar, Kayin and Kayah, where many communities have been exposed to the malaria volunteer model since 2015. The National Strategic Plan 2016–20 [10] plans to implement the malaria elimination interventions in these states in 2020, which further justifies the inclusion of the two states in the study.

All data collection tools (Additional file 2) were pilot tested with a sample of similar participants (one FGD with eight community members and one participatory workshop with five community leaders) and then modified in January 2018 before commencing data collection. The participants who joined the pilot testing activities were not recruited again for the actual research.

From January to March 2018, the first author purposively recruited eligible participants who were provided with information outlining the focus of the study and data collection methods (including measures to protect confidentiality), and participant’s role and responsibility in the study. This information was provided to the participants verbally and in a written information sheet (Additional file 3). Data saturation occurred after eight FGDs and two participatory workshops and then the researcher ceased recruitment in each subgroup.

A qualitative descriptive approach was used to explore the community’s knowledge and opinions on specific community-delivered models [19, 20]. The workshops and FGDs consisted of open-ended questions that allowed participants to reveal their opinions freely and interact with each other to build responses. Specific discussions in FGDs and participatory workshops focused on current malaria situation and priority health problems in the community, malaria control measures, available health services in the community, perspectives on the current malaria models, strategies to maintain the motivation and social role of malaria volunteers in the community, and the community preferred community-delivered model.

### Focus group discussions

The FGDs were held with community members and malaria volunteers in one township in Kayah (February 2018) and one in Kayin (March 2018) in secure locations, such as a private room in a church or in a community leader’s house. Each FGD lasted approximately one hour and were facilitated by the first author with the support of a research assistant, who was the note taker and interpreter. The FGDs were audio-recorded and field notes were taken. Prior to commencing the FGDs, the facilitator (first author) obtained informed consent from all participants, and collected non-identifying information such as age, sex and occupation.

### Participatory workshops

The two workshops were conducted in the field offices of Karuna Mission Social Soldieries, a faith-based local organization in Myanmar. Community leaders in each township in each state, typically one per village, participated in the workshops. The Kayah workshop took place in February 2018, and the Kayin workshop in March 2018 and each workshop lasted approximately eight hours. The first author facilitated the workshops with the assistance of a research assistant. Workshops were conducted in Burmese (the common language between community leaders and facilitators). A detailed agenda, including the roles and responsibilities of the facilitator (Additional file 2), was provided to participants prior to the commencement of the workshop. All participants provided informed consent and discussions in the workshop were audio-recorded and field notes were taken. Prior to commencing the workshops, the facilitators obtained non-identifying information from the participants relating to their role and responsibility in the community, age and residential township. The participants were grouped according to their occupation, geographical location and residential township and villages for brainstorming sessions that facilitated discussions and outcomes. Multiple techniques, such as preference ranking of health services, matrix scoring, and social and resource mapping were used in the workshops to stimulate participation and to generate rich data [21, 22].

Participants were provided with refreshments. Per diem of 4000 Kyat (approximately 2.5 USD) was given to each FGD participant and 10,000 Kyats (approximately 6.5 USD) to each workshop participant for their time involvement. In addition, their travel expenses were reimbursed.

The audio recordings were transcribed verbatim and field notes translated into English and prepared for analysis. Inductive thematic analysis [23] was used to analyse the qualitative data. This method was chosen because of the diverse opinions and views of the participants. The data analysis process included data immersion, coding, categorization/sub-theme development and major theme development [24]. Themes that emerged during data collection were captured and incorporated into the thematic framework developed during the data analysis stage. The level of analysis was mainly surface level and explored patterns and new understandings related to the perspectives and experiences of the participants. The first author analysed the data and second author randomly extracted 10% of the data and performed an independent thematic data analysis. Afterwards, the two authors discussed the themes and sub-themes and reached a consensus [25]. Codes were revised throughout data collection and analysis. All workshop participants were invited to undertake member checking [26] however only 16 participants did so.

## Results

### Community perceptions of malaria situation

The community perceived a rapid decline in the prevalence of malaria from 2014 to 2018. Before 2014 malaria was considered as a serious health problem. According to a malaria volunteer, “*We don’t see any shivering cases and sharing the blanket among febrile cases. Three years ago, there were one or two cases*.” (Villager, Kayah State). Although the community members were aware that malaria was no longer prevalent in their villages, they were concerned about the impact that malaria could have on their community if it returned and were still willing to be tested for malaria. Regular testing of malaria made community members feel safe, regardless of whether the test was positive or negative for malaria.

### Priority health problems in the community

**Fig. 1:**
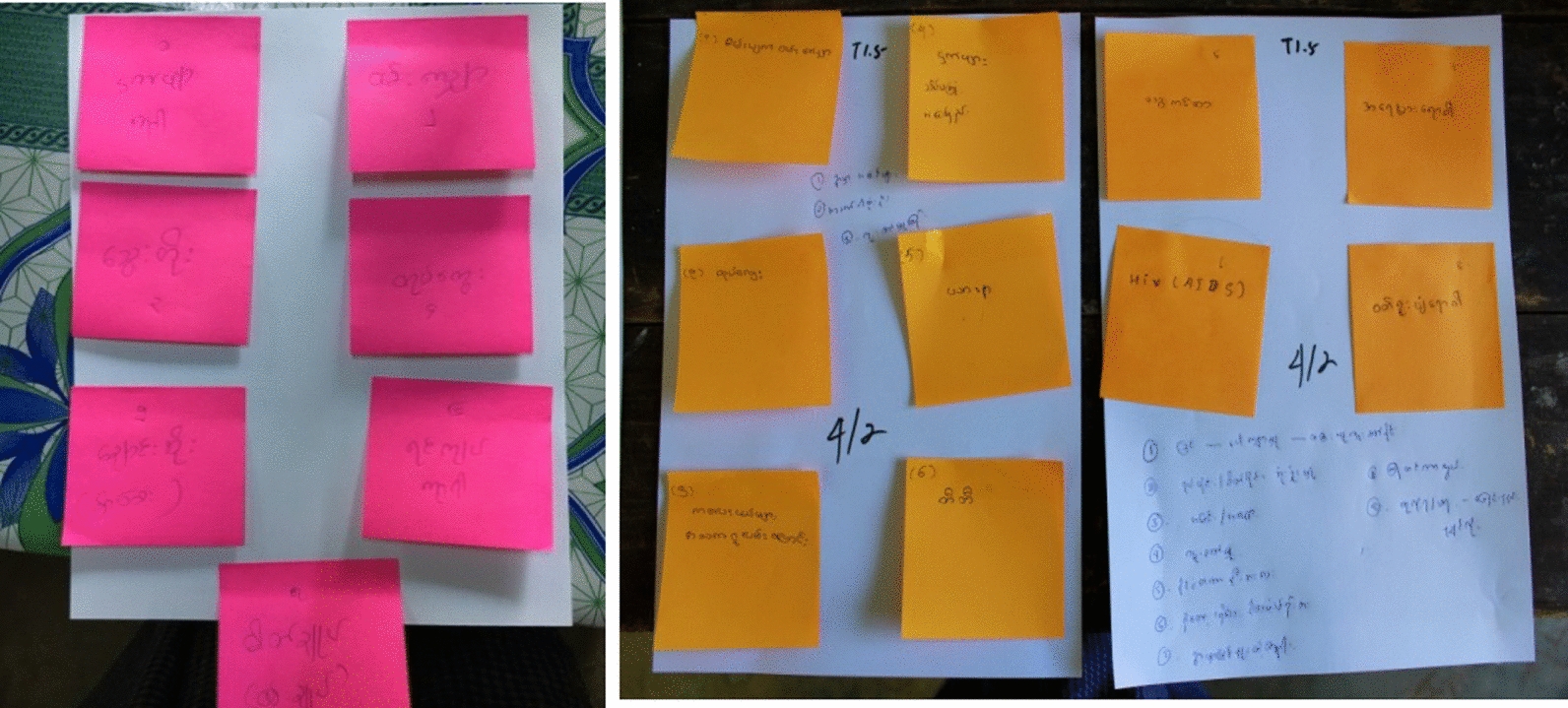
Village leaders’ priority list of health problems in the community (Left, in pink, Kayin State; right, in yellow, Kayah State.)

Apart from malaria, several priority health problems in the community were identified based on prevalence, severity and current incidence. These included communicable diseases such as the flu, diarrhoea, dengue, TB, worm infestation, rheumatic fever, measles, and non-communicable diseases such as hypertension and stroke (Fig. 1).

Among the non-malaria health problems listed, flu was ranked number one. Although accurate diagnosis of influenza virus infection is not possible in a village setting in Myanmar, fever, cough and sneezing were mentioned as the symptoms of flu.

- “We choose flu because it attacks everyone in the village. You can walk along this lane and ask every household, everyone suffers from fever. So it is important. It is more important than malaria.” (Villager, Kayah State).

Diarrhoea was the second priority, because it was common, severe and could be fatal, especially among children, and required urgent medical care.

- “Diarrhoea, because it is very fast, even within a day or even within hours, the patient can’t be saved. So serious” (Villager, Kayah State).

The community had concerns about poor access to quality health care for cases of severe diarrhoea. They considered that the unavailability of clean water was a root cause of diarrhoea in the villages. Dengue was listed as a third priority because of its serious impact on children and TB was considered the fourth priority because of its high transmissibility.

- “TB is also serious. If someone gets TB, it spreads in the village and so it is the main problem” (Malaria volunteer, Kayah State).

### Available health services in the community

**Fig. 2:**
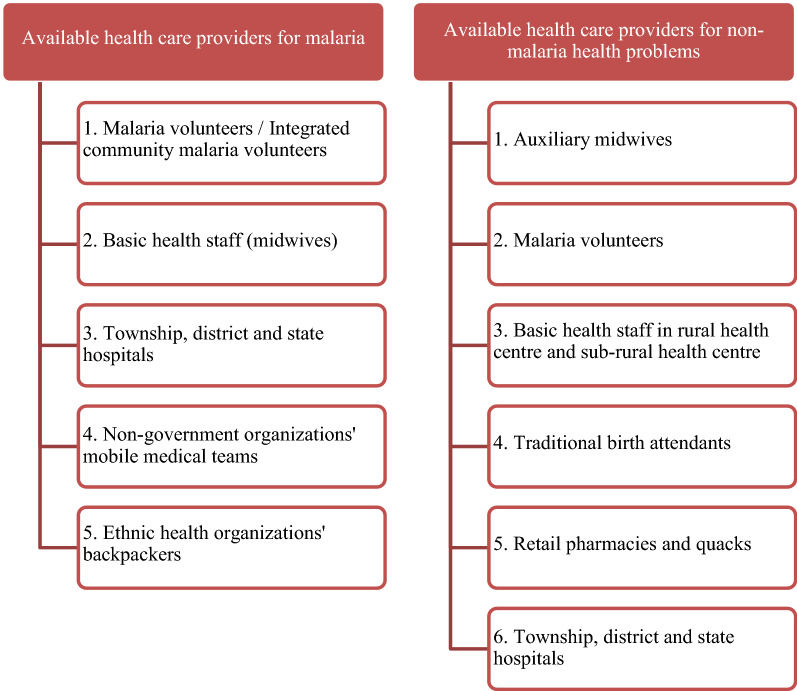
Available health care providers for malaria and non-malaria health problems in the community in decreasing order of community preference

There were multiple primary health care providers offering maternal and child health services and prevention activities for communicable diseases in the communities (Fig. 2). Ideally, one malaria volunteer is assigned for malaria and other provider(s), such as auxiliary midwives (AMWs), are trained to provide services for maternal and child health and other health problems.

The service coverage of one provider overlaps with other providers in some villages.

- “Me and my friend work together in this village for malaria. I work for [organization X] and she works for [organization Y]” (Malaria volunteer, Kayin State).

Malaria volunteers are quasi-formal health care providers and they diagnose, treat and prevent malaria in the community. They perform malaria diagnosis using rapid diagnostic test (RDT) and malaria treatment, and distribution of bed nets, repellent and health promotion materials. Community members were satisfied with the malaria services they received from malaria volunteers.

- “Firstly, we go to the provider in this village [the malaria volunteer] who can test for malaria. And then to Daw Rawk Khu [a village where a midwife lives]. We normally recover from the illness when we take medicines prescribed by him [the malaria volunteer].” (Villager, Kayah State).

The community members perceive that providing RDT is a routine task for a malaria volunteer. They consider that it is the responsibility of community members to actively seek testing to ensure that they remain free from malaria.

- “According to their [malaria volunteers’] job, they do blood testing once a month … then inform us whether we are positive or negative … if all the test results are negative, we are satisfied because we don’t have malaria…. And feel pleased” (Villager, Kayah State).

The community members said they want to keep malaria volunteers in their villages until a few years after malaria has been eliminated, because they were concerned that malaria might return. *“We need her to continue working as a malaria volunteer even after malaria has disappeared from this village. Who will take care of us when malaria comes back?”* (Villager, Kayin State).

They believe that having volunteers active in their village is essential to control a possible rebound in malaria.

- “Where do we get help when we get malaria? She is necessary. Even without malaria, she is still necessary. If malaria reappears, then we can do nothing if she is not here” (Villager, Kayah State).

The community considered that they receive higher quality malaria services, which includes malaria diagnosis and treatment from basic health staff (BHS) compared to malaria volunteers because the BHS have better facilities and staff have more knowledge about malaria. However, the community face barriers to treatment of malaria such as accessing BHS services in a timely manner, availability of BHS when they arrive at the facility, and increased direct and indirect transportation costs. Therefore, the community prefers to receive malaria services from malaria volunteers, who provide “acceptable” quality care and are located in their communities.

However, there were reports of a treatment service gap in villages; when a febrile person was RDT negative for malaria, volunteers cannot provide non-malaria treatment services. Sometimes, people with a negative RDT result do not go to the BHS when they are referred by the volunteer due to one or more access barriers. In these situations, people usually buy and take anti-malaria medicines from the local market. If their malaria symptoms are ameliorated after taking the medicines purchased from the market, the people lose trust in the RDT; these people purchase anti-malaria medications from the market when the situation occurs again.

- The test was negative but we had experience of malaria. When we received the blood test, there was no malaria. So, [the malaria volunteer] thought it [the sickness] was not because of malaria. But we believed it was. So, I bought anti-malaria drugs and took them, and then the disease was cured. (FGD, Kayin State).

In addition to malaria services, the community receives care for common health illnesses from the malaria volunteers. Although the volunteers are aware that they are not qualified doctor-level providers to treat all sorts of illnesses in the villages, they provide treatment services as the patients insist to get treatment from them rather than going to BHS.

- “Sometimes, I treat other diseases such as fever and cold. I know I am not a doctor, but I have to because the demand is there” (Malaria volunteer, Kayah State).

Community members also access primary health care from other health services such as local AMWs or they visit the rural health centre (RHC) or sub-rural health centre. Additionally, they receive services for malaria from local providers such as traditional birth attendants, pharmacies, and unqualified informal private providers (“quacks”).

The direct and indirect costs of receiving a service from BHS in an RHC were noted as a barrier to accessing RHC services. Consequently, people experiencing financial hardship generally seek non-malaria primary health care from either AMWs or traditional birth attendants, who are cheaper than the services provided at RHCs. Community members experiencing financial hardship also go to nearby pharmacies or quacks and buy medicines to treat their non-malaria illnesses.

The AMWs are the community’s first choice of health provider for non-malaria primary health care. *“In the village, people who don’t want to get services from sayarma *[*midwife*]*, the villagers who love and know her (AMW), go to her (AMW).”* (Villager, Kayah State). The most important factor that influences community members’ preference for a health provider is the quality of care provided, which includes the comprehensiveness of the services. The second factor is the time needed to travel to the service and availability of medicines and health providers when they arrive at the facility. The third factor is the convenience of transportation to and from the provider and finally the financial cost of accessing the health service. Additional factors noted by community members include language barriers and the provider’s degree of cultural and social integration in the community.

### Preferred community-delivered model

There was widespread agreement among community members, including leaders, that the community-delivered model should address as many common health problems as possible. “*If they *[*malaria volunteers*]* can manage other diseases, then we don’t have to go to other places to get health services”* (Villager, Kayah State). Malaria volunteers were aware of the community’s preference and were willing to provide services for other common health problems.

- “Yes, I know it [the community demand for non-malaria services]. And I am happy to expand my services as well. I have been treating other diseases with my own knowledge and if you [the facilitator] teach me how to handle those diseases, it would be excellent.” (Malaria volunteer, Kayin State).

The community considered that common health problems (other than malaria), that are within volunteers’ capacity to manage, should be integrated into the current malaria volunteer model.

- “It depends on them [malaria volunteers]. Which health activities that they want to do, and they can” (Village leader, Kayah State).

The other health problems that the community would like the malaria volunteers to address are flu (fever, cough and sneezing), diarrhoea, dengue, TB, maternal and child health, hypertension, headache and dizziness, in this order of priority.

However, community members acknowledged that it is not possible for a malaria volunteer to provide a full spectrum of services for all common diseases. They understood that the malaria volunteers can only provide prevention and referral services for other common diseases while at the same time providing full services for malaria.

- “Even though they [volunteers] cannot provide treatment for other diseases, if they know where we can get services and refer, that would be good. They are treating malaria, if they can also treat diarrhoea that would be fine” (Villager, Kayah State).

Furthermore, one malaria volunteer per village was considered not adequate to provide all the health services that the communities want and need. FGD participants requested two or more (up to five) volunteers per village to combat the many common diseases in the community 24 h a day, 7 days a week (24/7);

- “Actually, I think one volunteer alone is overburdened and so it should be at least two volunteers. One in the south and one in the north of the village as per groups of households” (Villager, Kayah State).

Malaria volunteers also supported this recommendation; “*It would be great if two volunteers work together for health care of this village*” (Malaria volunteer, Kayin State). The number of malaria volunteers required per village could be calculated based on the population and household size.

The FGD participants wanted to select volunteers based on the following criteria: education level, local residence, youth, having a volunteer spirit and the free time to work as a volunteer. Well-educated malaria volunteers can engage with the training content more fully and have greater capacity to apply their learning in the field. Permanent village residents are the communities’ preferred volunteers, as opposed to candidates from outside their villages, because a resident volunteer can provide services quickly and at low cost and is familiar with the village and its residents. The majority of FGD participants and all community leaders preferred young adult malaria volunteers, because they can travel easily to attend training and undertake field visits and are believed to have greater capacity to learn and to apply their knowledge. However, in one FGD, participants preferred older malaria volunteers because of their maturity and patience. Gender preference was not universal, as participants in one FGD suggested one female and one male volunteer per village, because they could address gender-sensitive health issues such as reproductive health and men’s health. However, all other FGD participants agreed that the gender of malaria volunteers did not matter. Finally, participants acknowledged that it would be difficult to recruit “ideal” volunteers, due to limited human resources in the villages.

The FGD participants wanted to receive primary health care services for common health problems in the village 24/7, and to receive the same quality of service from each volunteer. To fulfil this need, volunteers will need training and support. Training of malaria volunteers for multiple common health problems will give the community a greater choice of provider. Multiple volunteers in the same community could provide technical support and share medical commodities that will prevent shortage of commodities.

However, there were differences of opinion among FGD participants who believed that it would be inappropriate to train and assign volunteers according to particular health problems, such as one volunteer for malaria and another volunteer for maternal and child health.

- “One (person) may suffer many diseases and so need holistic care” (Villager, Kayah State). “They [volunteers] should have the same level of knowledge. One can replace another when he or she is absent” (Villager, Kayah State).

They envisaged no major causes of conflict between volunteers, although two or more volunteers would be providing the same package of services in the same village. To prevent any conflicts, community leaders should facilitate the volunteers’ assignments and one volunteer would provide backup to ensure continuous coverage of services if another is absent. The community leaders agreed to manage conflict between volunteers to the best of their capacity.

## Discussion

The malaria volunteers’ role is changing as Myanmar transitions from a malaria control to elimination programme and towards the goal of universal health coverage. Our qualitative findings demonstrated that community members and leaders, as well as malaria volunteers, were aware that clinical malaria burden had declined dramatically in their area, but they still regarded malaria as important because of the profound impact it had on their communities in the past. Additionally, they were concerned about other communicable diseases such as flu, diarrhoea, dengue and TB. The FGD participants identified malaria volunteers as the first point of contact for malaria services and AMWs as the main providers of non-malaria health services. Community members and leaders appreciated the value of the health services provided by both. Although malaria volunteers were seen to be providing valuable health services, the community members and leaders suggested several ways to improve volunteers’ role and motivation. They identified their preferred service model, that is, an integrated community-delivered model that provides a full spectrum of services for malaria and covers common health problems in the community along with prevention and assisted referral services 24/7. Despite the significant input that community members and leaders made to the design of an integrated community-delivered malaria elimination model, these findings may change over time and according to geographical area. Nevertheless, an evidence-based, community preferred, pragmatic community-delivered integrated model should be constructed based on the context of malaria elimination and progressing towards universal health coverage in Myanmar.

### Malaria, its consequences and malaria elimination

In terms of awareness on malaria, the communities who participated in this study expect most RDT results for malaria to be negative but remained willing to be tested because they were concerned about the consequences of the disease. However, these residual concerns about clinical malaria may change over time, as the epidemiology of RDT-detectable malaria changes. Knowledge, attitudes, and practices related to clinical malaria may need to be monitored to enable appropriate behaviour change communication around the importance of maintaining high testing rates in low RDT-detectable malaria prevalence environments in Myanmar.

Aside from RDT-detectable clinical malaria, undetected subclinical malaria may sustain malaria transmission in the population [27–29]. A national malaria surveillance system is comprehensive only when it can detect both clinical and subclinical malaria cases. It is critical to detect and treat both clinical and subclinical malaria in order to achieve malaria elimination in a region [28, 30, 31]. Importantly, community members, leaders and volunteers did not understand the concept of subclinical malaria although the facilitators probed to discuss the importance of subclinical malaria during data collection, and therefore it was not discussed in any FGDs and workshops. This finding echoes a study conducted in Laos, which found that about half of the study population disagreed that a seemingly healthy person could have malaria parasites in their blood [32]. Collectively, these findings highlight the importance of community health literacy [33] on crucial malaria elimination concepts for the success of malaria elimination programmes in the GMS.

Community members and leaders preferred health services provided by malaria volunteers and AMWs, which confirms that these roles are still popular and valued by the community. However, there is a gap between community expectations of health service providers and the available health services provided by malaria volunteers in the community. The malaria volunteer model was developed decades ago in the context of high malaria prevalence in rural areas in Myanmar where volunteers could pinpoint their services on malaria. Over the decades, malaria epidemiology and the context of primary health care in Myanmar has already changed and therefore adaption of the malaria volunteer model is unavoidable. Acknowledging this demand as the country moved towards malaria elimination, the Ministry of Health and Sports recently transformed malaria volunteers into ICMVs (delivering services for malaria, dengue, lymphatic filariasis, TB, HIV/AIDS and leprosy); nevertheless, the transition process did not integrate community voices. Therefore, the next adaptation needs to apply an all-inclusive approach—the only way that could maintain the popularity and usefulness of the community-delivered model in the malaria elimination programme—integrating inputs from international evidence, community members and stakeholders.

### The community-proposed integrated community-delivered model for Myanmar

The community voices in this study said that their preferred service from volunteers would include prevention, diagnosis and treatment of common diseases in the community associated with fever. Incorporation of a comprehensive curative and preventive service package for fever, even when the RDT result was negative would satisfy community members and leaders; while community members and leaders were not disappointed by negative RDTs for malaria, they were unhappy with the end-of-service outcomes after receiving negative RDT results. Globally, the implementation of community-delivered models had no significant impact on fever prevalence, which might be due to restrictions of volunteer services to malaria only [2]. To provide a combination of services for malaria, RDT-negative fever, diarrhoea, TB and dengue, prioritizing community demand and local epidemiology, an evidence-based community-delivered integrated malaria elimination model is yet to be developed.

In the proposed model, community members specified two or more volunteers per village (ranging from two to five), to provide services for the many common diseases in the community 24/7. Studies implemented in Africa found that malaria volunteers were overburdened and disrupted due to their routine life [34–36] and their workload must be shared in order to maintain the volunteer model’s effectiveness. This would support increasing the number of volunteers per village, however any increases in volunteer numbers must be balanced with the increased cost of adding more volunteers to the national malaria programme.

The proposed integrated model will contribute to achieving universal health coverage in Myanmar. In addition to achieving malaria elimination in Myanmar by 2030, the government has set the target of achieving universal health coverage by 2030 [37]. Three key dimensions of universal health coverage are; essential health service coverage, financial risk protection, and equity in coverage [38]. The proposed integrated community-delivered model will contribute to a higher level of essential health service coverage [39]. Myanmar has over 60,000 villages and currently over 15,000 are serviced by malaria volunteers/ICMVs [40]. If they transform into the proposed integrated volunteers, they will play a major role in the provision of essential health services in the rural setting of Myanmar.

### The integrated community-delivered model for GMS countries

Not only Myanmar, but other GMS countries (except Yunnan Province in China) are also implementing the malaria volunteer model. These GMS countries are considering redesigning the current model in response to changing malaria epidemiology, community demands and national malaria elimination strategies. The GMS Civil Society Organization Platform endorsed a resolution at the Regional Workshop on Disease Integration to integrate interventions for common diseases into the malaria volunteer model [41]. It is recommended that community consultations should be repeated in other GMS countries in order to support the development of country-specific evidence-based integrated models in GMS countries.

## Strengths and limitations

The study employed a participatory research model to engage community members and leaders, and malaria volunteers to provide a wide range of views on the preferred model. However, contextual factors, particularly time and geographical location, are important in the translation and application of the qualitative findings from this study. Firstly, the data were collected during a year in which malaria had largely disappeared as a major communicable disease and before the roll out of ICMV model in those areas. Similarly, the epidemiology of other communicable diseases such as flu in the rural areas of Myanmar is changing. The community’s opinions may differ if they are collected at another point of time. For example, the community members and leaders mentioned that malaria, flu, diarrhoea, dengue and TB were common and were diseases that the community was concerned about. The data was collected during the flu season, which may have increased community members’ focus on flu. If data collection occurred at another time of year, then the community’s priority list might have been different.

Secondly, the community consultations were conducted in two states of Myanmar due to limited resources. The perspectives of community members and leaders in these two states may not represent the perspectives of community members and leaders in other states and regions of Myanmar. The findings from this study have limited application to future model constructions; nationwide community and stakeholder consultations should be done ahead of future model development to ensure national representativeness.

Methodologically, the original plan was to avoid mixing current and ex-malaria volunteers (volunteer villagers) with ordinary community members (other villagers) in FGDs. However, volunteers did not identify themselves on purpose and they joined the FGDs as ordinary villagers. They provided expert opinions in FGDs, and only then did the facilitator realize that they might be ex-volunteers or current volunteers. Some were currently working for implementing partners in Myanmar and some had retired from volunteer work, so chose not to identify themselves as malaria volunteers. Nonetheless, mixing volunteers and other villagers supported the diversity of participants in the FGDs and assisted in exploring different opinions about health service provision. Overall, mixing volunteers and other villagers in the same FGDs had minimal impact on the analysis and reporting. Each theme was analysed and reported from the perspectives of community members, leaders and volunteers, encompassing the opinions of a diverse group of community members and authorities.

Although assumption bias may contaminate the qualitative findings, triangulating data from the community members, leaders and volunteers, and then conducting an inductive thematic analysis of the qualitative data minimized it. This was mitigated by reading widely and repeatedly in the field, across disciplines, comparing interpretations with the results of other studies (whenever possible), both substantive and theoretical in the writing up phase [23].

## Conclusions and recommendations

The community suggested their preferred community-delivered model, which is an integrated community-delivered model that provides a full spectrum of services for five communicable diseases. However, the suggested community-delivered model might not be feasible to implement in full scale in the context of Myanmar.

Therefore, multiple stakeholders’ voices need to be considered in the development of a future community-delivered integrated malaria elimination model for Myanmar. Consultations with health stakeholders from Myanmar Ministry of Health and Sports and implementing partners are recommended to capture their opinions on community-delivered models. Once completed and both perspectives are considered, a community-delivered integrated malaria elimination model should be developed which balances the needs of the community and other stakeholders with available resources and considers the current health policies in Myanmar. The developed model should be pilot tested in Myanmar and considered for national scale-up. The model needs to be reviewed and revised periodically to reflect the changing epidemiology of diseases in rural areas and the dynamic political context of the country. Beyond Myanmar, qualitative consultations on community-delivered models in other GMS countries should be implemented to develop country-specific integrated community-delivered models tailored for the elimination context.

## Supplementary Information

**Additional file 1:** The diseases included in the Integrated Community Malaria Volunteer (ICMV) model and interventions ICMVs provide.

**Additional file 2:** Qualitative research data collection tools.

**Additional file 3.** Informed consent forms.

**Additional file 4.** Approval letters from Myanmar Ministry of Health and Sports and the Data Tranfer Agreement.

**Additional file 5.** Ethics Review Committee Certificates of approval.

## Data Availability

The datasets generated and/or analysed during the current study are not publicly available as the study collected data from specific townships and villages in Myanmar, and the information may be identifiable to particular individuals, risking a breach in confidentiality; but are available from the corresponding author on reasonable request.

## Abbreviations

AMW: Auxiliary Midwife
BHS: Basic Health Staff
FGD: Focus Group Discussion
GMS: Greater Mekong Sub-region
HIV/AIDS: Human Immunodeficiency Virus/Acquired ImmunoDeficiency Syndrome
ICMV: Integrated Community Malaria Volunteer
RDT: Rapid Diagnostic Test
RHC: Rural Health Centre
TB: Tuberculosis
