# Supplementary material for: Community demand for comprehensive primary health care from malaria volunteers in South-East Myanmar: a qualitative study
Source: Malar J. 2021 Jan 6;20:19. doi: 10.1186/s12936-020-03555-4 (PMC7789746; doi:10.1186/s12936-020-03555-4)
Supplement: Supplementary file 1 — Additional file 1: The diseases included in the Integrated Community Malaria Volunteer (ICMV) model and interventions ICMVs provide. [file 12936_2020_3555_MOESM1_ESM.docx]

The diseases included in the Integrated Community Malaria Volunteer (ICMV) model and interventions ICMVs provide

|  | Disease | Interventions |
| --- | --- | --- |
| 1 | Malaria | - Prevention and health education, community mobilisation for malaria activities - Helping in distribution of long-lasting insecticidal nets and dipping existing bed nets - Early diagnosis, treatment and referral of malaria cases according to the National Malaria Treatment Guidelines - Early warning and reporting of possible malaria outbreaks in the community to the health department - Data entry, compilation and reporting of rapid diagnostic test-tested malaria cases using the prescribed formats - Helping in entomological, malaria elimination and community-based research activities |
| 2 | Dengue | - Assisting the Vector Borne Diseases Control Programme staff and basic health staff (BHS) in vector control activities - Helping in referral of dengue suspected patients to the nearest health centre |
| 3 | Lymphatic filariasis | - Helping BHS in mass drug administration activity for lymphatic filariasis elimination - Reporting of lymphatic filariasis cases to the health department and assisting in the home-based care of lymphatic filariasis cases |
| 4 | Tuberculosis (TB) | - Checking for TB signs and symptoms, and referral of suspected TB patients - Contact tracing of TB patients in their communities - Serving as Directly Observed Treatment providers - Following up the lost-to-follow-up TB patients (defaulter tracing) - Helping TB patients in follow-up sputum examinations - Assisting BHS in TB health education talks and active case detection activities |
| 5 | HIV/ AIDS | - Providing health education on HIV/AIDS and other sexually transmitted diseases (STDs) - Assisting in the mitigation of discrimination against HIV/AIDS patients - Informing villagers of locations of clinics where they can get free services for HIV/AIDS and other STDs - Helping in referral of clients who need STD treatment and HIV testing   (Note: ICMVs must keep HIV/AIDS and STD information confidential.) |
| 6 | Leprosy | - Providing health education in the community – communicating key leprosy messages to villagers - Referral of suspected leprosy cases to health departments - Referral of disabled, old and new leprosy patients who are suffering from reaction and complications of leprosy - Assisting BHS and leprosy program staff to detect new leprosy cases - Assisting the leprosy program in its public health projects |
