# Supplementary material for: Community demand for comprehensive primary health care from malaria volunteers in South-East Myanmar: a qualitative study
Source: Malar J. 2021 Jan 6;20:19. doi: 10.1186/s12936-020-03555-4 (PMC7789746; doi:10.1186/s12936-020-03555-4)
Supplement: Supplementary file 2 — Additional file 2: Qualitative research data collection tools. [file 12936_2020_3555_MOESM2_ESM.docx]

**Qualitative research data collection tools before pretesting**

**Participatory workshop guide**

**Responsible persons**

Main Facilitator (MF) (PI Win Han Oo) – lead the overall process and facilitate to get the enrich data in ethical approach

Additional facilitator 1 (F1) – Note taking, audio recording and supplementary facilitation

Additional facilitator 2 (F2) (optional) – logistic, administrative, financial managements and supplementary facilitation, translation (if facilitator 2 is not involved, main facilitator and facilitator 1 will share the responsibilities of facilitator 2)

**Agenda**

| **No** | **Session** | **Time** | | **Facilitator (F)** |
| --- | --- | --- | --- | --- |
| 1 | Introduction and informed consent | 9:00 | 9:30 | MF |
| 2 | Introduction of malaria, situation and interventions of malaria in Myanmar | 9:30 | 10:00 | MF |
|  | Tea break | 10:00 | 10:15 |  |
| 3 | Theme 1 small groups discussion (preference ranking or matrix scoring) applying questions and probes and 5 minutes presentations | 10:15 | 11:00 | MF, F1 and F2 |
| 4 | Theme 2 small groups discussion (diagramming and visualization – social maps) applying questions and probes and 5 minutes presentations | 11:00 | 11:45 | MF, F1 and F2 |
| 5 | Theme 3 small groups discussion (semi-structure interview) applying questions and probes and 5 minutes presentations | 11:45 | 12:30 | MF, F1 and F2 |
|  | Lunch break | 12:30 | 13:30 |  |
| 6 | Theme 4 small groups discussion (diagramming and visualization – social maps) applying questions and probes and 5 minutes presentations | 13:30 | 14:15 | MF, F1 and F2 |
| 7 | Theme 5 small groups discussion (diagramming and visualization – resource maps) applying questions and probes and 5 minutes presentations | 14:15 | 15:00 | MF, F1 and F2 |
|  | Tea break | 15:00 | 15:15 |  |
| 8 | Theme 6 small groups discussion (preference ranking) applying questions and probes and 5 minutes presentations | 15:15 | 16:00 | MF, F1 and F2 |
| 9 | Theme 7 small groups discussion (diagramming and visualization – mind maps) applying questions and probes and 5 minutes presentations | 16:00 | 16:45 | MF, F1 and F2 |
| 10 | Conclusion and group recommendations | 16:45 | 17:00 | MF |

**Theme, questions and probes**

| **No** | **Themes** | **Questions and probes** |
| --- | --- | --- |
| 1 | Current malaria situation and priority health problems in the respective community | - Has your village/community ever experience malaria? - If yes, how was it impacted on your village/community? - How did you manage to help the patients? - Do you think malaria is still a priority health issue in your village/community   - - Why do you think so? - What are the other health problems in your village/community   - - How do you rank them? |
| 2 | Malaria control measures and available health services in the respective community | - What are the sources of health care services for your community?   - - Which sources do you prefer and why?     - Which sources do you not prefer and why? - What are the available services/interventions for malaria in your community?   - - Do you think are they enough to compact malaria?     - If not, what interventions are you going to suggest? - Where does your community get the malaria interventions/services recently?   - - Do you think is it suitable?     - If not, why and what’s your suggestion to meet the needs? |
| 3 | Views and perspectives on the current malaria CHW models | - Have you ever received services from the CHW in your village/community?   - - If yes, tell me your story/experience     - If no, can you tell me other people’s experiences in your village? - Do you think current CHW in your village/community is effective to control malaria?   - - Why? And How? |
| 4 | Policy and strategic barriers and enablers for effective malaria control and elimination in their community | - Have you ever noticed that the local policy, rules and regulations caused barriers and/or enablers for the malaria interventions in the community?   - - What are they?     - How did they affected the interventions     - How do we enhance the enablers in the future?     - How do we overcome the barriers in the future? |
| 5 | Available community supports for malaria control and elimination in their community | - Did the community support the CHW in the past for malaria activities?   - - How did they support? - Do you think the CHW should get local supports from the community in the future?   - If yes, why?     - What supports they should receive to effectively work for the community?     - How can the community support them?   - If no, why?     - Do you think they will survive without community support? |
| 6 | Strategies to maintain the motivation and social role of CHWs in the community | - What is your opinion on health care and social role of the CHW in your village/community?   - - Was it declining or increasing compared to the past 2-3 years?     - Why do you think it was happening so?     - How has the CHW responded it? - Do you think is it necessary to maintain/uplift the role of CHW in the village/community? - How do we maintain them?   - - Can you elaborate each strategy in detail? |
| 7 | Culture, customs and norms of the ethnic communities that play as barriers and enablers for effective malaria control and elimination in their community | - Have you ever noticed that the local culture, customs and norms of the ethnic communities caused barriers and/or enablers for the malaria interventions in the community?   - - What are they?     - How did they affect the interventions?     - How do we enhance the enablers in the future?     - How do we overcome the barriers in the future? |

**Focus Group Discussion (FGD) guide**

**Introduction**

Now that I have explained the purpose of our study and you have all agreed to participate, can we begin the discussion?

Theme, questions and probes

| **No** | **Themes** | **Questions and probes** |
| --- | --- | --- |
| 1 | Current malaria situation and priority health problems in your community | - Do you know what malaria is?   - - Have you or your family ever experienced malaria?     - If yes, how was it impacted on you / your family?     - If no, have you ever heard of anyone in your village/community suffered from malaria? - Do you think malaria is still a priority health issue in your village/community   - - Why do you think so? - What are the other health problems in your village/community   - - How do you rank them? |
| 2 | Malaria control measures and available health services in the respective community | - What are the sources of health care services for your community?   - - Which sources do you prefer and why?     - Which sources do you not prefer and why? - What are the available services/interventions for malaria in this community?   - - Do you use them, when? How have they worked?     - What do you think about the current malaria services in your community? |
| 3 | Views and perspectives on the current malaria CHW models | - Have you ever received services from the CHW in your village/community?   - - If yes, tell me your story/experience     - If no, can you tell me other people’s experiences around you? |
| 4 | Available community supports for malaria control and elimination in their community | - What supports did the CHW receive from the community for malaria activities?   - - How did the community support? - Can you explore the reasons for the CHWs why they should or shouldn’t get local supports from the community?   - - What supports should they receive to effectively work for the community?     - How can the community support them?     - If you think the community shouldn’t support them, why? |
| 5 | Strategies to maintain the motivation and social role of CHWs in the community | - What is your opinion on health care and social role of the CHW in your village/community?   - - Was it declining or increasing compared to the past 2-3 years?     - Why do you think it was happening so?     - How has the CHW responded to it? - What is your opinion on maintaining/uplifting the role of CHW in the village/community? - How do we maintain them?   - - Can you elaborate each strategy in detail? |
| 6 | Culture, customs and norms of the ethnic communities that play as barriers and enablers for effective malaria control and elimination in their community | - Are there any barriers or enablers to people in the community accessing the malaria interventions?   - - What are they? Local customs, experiences?     - How did they affect the interventions?     - How do we enhance the enablers in the future?     - How do we overcome the barriers in the future? |

This is the end of my questions. Do you have anything else you would like to say about this project?

Do you have any questions for me?

Thank you very much for participating.

**End of session**

**Qualitative research data collection tools after pretesting**

**Participatory workshop guide**

**Responsible persons**

Main Facilitator (MF) (PI Win Han Oo) – lead the overall process and facilitate to get the enrich data in ethical approach

Additional facilitator 1 (F1) – Note taking, audio recording and supplementary facilitation

Additional facilitator 2 (F2) (optional) – logistic, administrative, financial managements and supplementary facilitation, translation (if facilitator 2 is not involved, main facilitator and facilitator 1 will share the responsibilities of facilitator 2)

**Agenda**

| **No** | **Session** | **Time** | | **Facilitator (F)** |
| --- | --- | --- | --- | --- |
| 1 | Introduction and informed consent | 9:00 | 9:30 | MF |
| 2 | Introduction of malaria, situation and interventions of malaria in Myanmar | 9:30 | 10:00 | MF |
|  | Tea break | 10:00 | 10:15 |  |
| 3 | Theme 1 small groups discussion (preference ranking or matrix scoring) applying questions and probes and 5 minutes presentations | 10:15 | 11:00 | MF, F1 and F2 |
| 4 | Theme 2 small groups discussion (diagramming and visualization – social maps) applying questions and probes and 5 minutes presentations | 11:00 | 11:45 | MF, F1 and F2 |
| 5 | Theme 3 small groups discussion (semi-structure interview) applying questions and probes and 5 minutes presentations | 11:45 | 12:30 | MF, F1 and F2 |
|  | Lunch break | 12:30 | 13:30 |  |
| 6 | Theme 4 small groups discussion (diagramming and visualization – social maps) applying questions and probes and 5 minutes presentations | 13:30 | 14:15 | MF, F1 and F2 |
| 7 | Theme 5 small groups discussion (diagramming and visualization – resource maps) applying questions and probes and 5 minutes presentations | 14:15 | 15:00 | MF, F1 and F2 |
|  | Tea break | 15:00 | 15:15 |  |
| 8 | Theme 6 small groups discussion (preference ranking) applying questions and probes and 5 minutes presentations | 15:15 | 16:00 | MF, F1 and F2 |
| 9 | Theme 7 small groups discussion (diagramming and visualization – mind maps) applying questions and probes and 5 minutes presentations | 16:00 | 16:45 | MF, F1 and F2 |
| 10 | Conclusion and group recommendations | 16:45 | 17:00 | MF |

**Theme, questions and probes**

| **No** | **Themes** | **Questions and probes** |
| --- | --- | --- |
| 1 | Current malaria situation and priority health problems in the respective community | - Has your village/community ever experience malaria? - If yes, how was it impacted on your village/community? - How did you manage to help the patients? - Do you think malaria is still a priority health issue in your village/community   - - Why do you think so? - What are the other health problems in your village/community   - - How do you rank them? |
| 2 | Malaria control measures and available health services in the respective community | - What are the sources of health care services for your community?   - - Which sources do you prefer and why?     - Which sources do you not prefer and why? - What are the available services/interventions for malaria in your community?   - - Do you think are they enough to compact malaria?     - If not, what interventions are you going to suggest? - Where does your community get the malaria interventions/services recently?   - - Do you think is it suitable?     - If not, why and what’s your suggestion to meet the needs? |
| 3 | Views and perspectives on the current malaria CHW models | - Have you ever received services from the CHW in your village/community?   - - If yes, tell me your story/experience     - If no, can you tell me other people’s experiences in your village? - Do you think current CHW in your village/community is effective to control malaria?   - - Why? And How? |
| 4 | Policy and strategic barriers and enablers for effective malaria control and elimination in their community | - Have you ever noticed that the local policy, rules and regulations caused barriers and/or enablers for the malaria interventions in the community?   - - What are they?     - How did they affected the interventions     - How do we enhance the enablers in the future?     - How do we overcome the barriers in the future? |
| 5 | Available community supports for malaria control and elimination in their community | - Did the community support the CHW in the past for malaria activities?   - - How did they support? - Do you think the CHW should get local supports from the community in the future?   - If yes, why?     - What supports they should receive to effectively work for the community?     - How can the community support them?   - If no, why?     - Do you think they will survive without community support? |
| 6 | Strategies to maintain the motivation and social role of CHWs in the community | - What is your opinion on health care and social role of the CHW in your village/community?   - - Was it declining or increasing compared to the past 2-3 years?     - Why do you think it was happening so?     - How has the CHW responded it? - Do you think is it necessary to maintain/uplift the role of CHW in the village/community? - How do we maintain them?   - - Can you elaborate each strategy in detail? |
| 7 | Culture, customs and norms of the ethnic communities that play as barriers and enablers for effective malaria control and elimination in their community | - Have you ever noticed that the local culture, customs and norms of the ethnic communities caused barriers and/or enablers for the malaria interventions in the community?   - - What are they?     - How did they affect the interventions?     - How do we enhance the enablers in the future?     - How do we overcome the barriers in the future? |

**Focus Group Discussion (FGD) guide**

**Introduction**

Now that I have explained the purpose of our study and you have all agreed to participate, can we begin the discussion?

Theme, questions and probes

| **No** | **Themes** | **Questions and probes** |
| --- | --- | --- |
| 1 | Current malaria situation and priority health problems in your community | - Do you know what malaria is?   - - Have you or your family ever experienced malaria?     - If yes, how was it impacted on you / your family?     - If no, have you ever heard of anyone in your village/community suffered from malaria? - Do you think malaria is still a priority health issue in your village/community   - - Why do you think so? - What are the other health problems in your village/community   - - How do you rank them? |
| 2 | Malaria control measures and available health services in the respective community | - What are the sources of health care services for your community?   - - Which sources do you prefer and why?     - Which sources do you not prefer and why? - What are the available services/interventions for malaria in this community?   - - Do you use them, when? How have they worked?     - What do you think about the current malaria services in your community? |
| 3 | Views and perspectives on the current malaria CHW models | - Have you ever received services from the CHW in your village/community?   - - If yes, tell me your story/experience     - If no, can you tell me other people’s experiences around you? |
| 4 | Available community supports for malaria control and elimination in their community | - What supports did the CHW receive from the community for malaria activities?   - - How did the community support? - Can you explore the reasons for the CHWs why they should or shouldn’t get local supports from the community?   - - What supports should they receive to effectively work for the community?     - How can the community support them?     - If you think the community shouldn’t support them, why? |
| 5 | Strategies to maintain the motivation and social role of CHWs in the community | - What is your opinion on health care and social role of the CHW in your village/community?   - - Was it declining or increasing compared to the past 2-3 years?     - Why do you think it was happening so?     - How has the CHW responded to it? - What is your opinion on maintaining/uplifting the role of CHW in the village/community? - How do we maintain them?   - - Can you elaborate each strategy in detail? - Do you think 1 CHW / village is enough, why? - How would you like to assign CHW for different common health problems in your village? Give reason for your suggestion. - Do government health staff recognize CHW and their role? |
| 6 | Culture, customs and norms of the ethnic communities that play as barriers and enablers for effective malaria control and elimination in their community | - Are there any barriers or enablers to people in the community accessing the malaria interventions?   - - What are they? Local customs, experiences?     - How did they affect the interventions?     - How do we enhance the enablers in the future?     - How do we overcome the barriers in the future? |

This is the end of my questions. Do you have anything else you would like to say about this project?

Do you have any questions for me?

Thank you very much for your participation.

**End of session**
