# Supplementary material for: Community demand for comprehensive primary health care from malaria volunteers in South-East Myanmar: a qualitative study
Source: Malar J. 2021 Jan 6;20:19. doi: 10.1186/s12936-020-03555-4 (PMC7789746; doi:10.1186/s12936-020-03555-4)
Supplement: Supplementary file 3 — Additional file 3. Informed consent forms. [file 12936_2020_3555_MOESM3_ESM.pdf]

Ethics Review Committee
Department of Medical Research
Ministry of Health and Sports
Republic of the Union of Myanmar

Informed Consent Form for "Perspectives of community delivered models for the malaria elimination in Myanmar: A qualitative study", Focus Group Discussion (FGD) with community members

This informed consent form is for **community members**, invited to participate in the **"Perspectives of community delivered models for the malaria elimination in Myanmar: A qualitative study".**

**Name of Principal Investigator:**	Dr. Win Han Oo
**Name of Organization:**	Deakin University and Burnet Institute
**Name of sponsor:**	Deakin University and Burnet Institute
**Title of study:**	"Perspectives of community delivered models for the malaria elimination in Myanmar: A qualitative study"

PART 1: Information Sheet

Introduction

I am ______________ and I work for the Burnet Institute funded project in Myanmar. I am conducting a study on "Perspectives of community delivered models for the malaria elimination in Myanmar: A qualitative study". This consent form will provide you with some information about the study. It may contain words that you do not understand. Please ask me to explain any words or information that you do not clearly understand as we go through the form. I will also give you a signed copy (or unsigned, if you wish) to keep for your record.

Purpose of the research

This study is being conducted by Dr Win Han Oo for his PhD degree at Deakin University Australia, and is funded by Deakin University and Burnet Institute. Myanmar has the highest burden of malaria in the Greater Mekong Sub-region and aims to eliminate malaria by 2030. The PhD project aims to develop a community-delivered malaria elimination model that is acceptable, operational, pragmatic, evidence based and effective compared to the traditional model for malaria elimination in Myanmar.

This study aims to explore the perspectives, views and inputs of key health stakeholders and community members in Myanmar on community-delivered models for the development of community- delivered malaria elimination model. We are doing research to:

- explore the views and perspectives on the current malaria CHW models
- explore the strategies to maintain the motivation and social role of CHWs in the community
- explore the factors that need to be addressed during the transition from malaria control to elimination setting focusing on the community delivered models

We are collecting information from health staff, malaria implementing partner staff, community leaders and community members.

Type of research intervention

You are invited to participate in a Focus Group Discussion (FGD) as part of this study. In this focus group, you will be discussing the malaria services in your community with your fellow community members and will be facilitated by a research team member.

Participant selection

You are being invited to take part in this research because we feel that your experience and knowledge as a community member can contribute much to our understanding and knowledge of community delivered models for malaria elimination in Myanmar.

Voluntary Participation

Your participation is voluntary. It is your right to decide whether or not you want to join the study or to stop participating at any time. You are not required to answer any questions that you are not comfortable with. Whether you choose to participate in this study or not, and any information you choose to provide to us, will not affect the healthcare you receive in any way.

Procedure

The Focus Group Discussion will include 6-8 community members and will be conducted by one facilitator and one note taker. The discussion will be held in a place where other community members cannot hear the discussion. The topics will be discussed with the same sex participants of you. We will be taking notes and an audio recording of this Focus Group Discussion. Your name will not be recorded in either the written notes or the audio recording.

Prior to commencing the FGD, the facilitator will obtain non-identifying information relating to your role/responsibility in the community, age, sex and occupation. We will be discussing the current malaria situation and priority health problems, malaria control measures and available health services in your community, your views and perspectives on the current malaria CHW models, available community supports for malaria control and elimination in the community, strategies to maintain the motivation and social role of CHWs in the community and culture, customs and norms of the ethnic communities that act as barriers and enablers for effective malaria control and elimination in your community.

Duration

The Focus Group Discussion will take approximately one to two hours.

Confidentiality

Information obtained in this study will be kept confidential and will not be shared with anyone outside the study team. Your name or other identifying information will not appear in the notes from this discussion or in the final report, and only staff participating in the study will have access to the information you provide. Once you agree to participate in the study, we will ask you to choose a pseudonym (a fake name) in order to try to protect your privacy throughout your participation.

All consent forms, audio recordings, and notes from this study will be stored in a locked filing cabinet, and only study staff will have access to them. Representatives of "The Alfred Office of Ethics & Research Governance" and "Department of Medical Research Ethics Review Committee" will be able to access these documents. However they would only do this to ensure that your privacy is being maintained and protected. We will use the audio recordings to produce a written record of our conversation. The audio recordings will then be destroyed after they have been copied to a password protected computer. The written record and any notes from this discussion will be stored securely for a period of seven years and then destroyed.

Risk and Discomforts

During the course of the Focus Group Discussion, you will be asked to discuss your experiences and opinions on malaria services and malaria elimination in your community. . There is a small risk that you may feel uncomfortable answering these questions. However, we do not wish this to happen, and you may refuse to answer any question or not take part in the study if you feel uncomfortable answering any question(s).

Benefits

You will not get any direct benefit from participating in this study; however, your participation in this study will help the principal investigator Dr Win Han Oo create the community-delivered malaria elimination model that is acceptable, operational, pragmatic, evidence based and effective compared to the traditional model for malaria elimination in Myanmar.

Incentives

We will provide refreshment, 4000 Kyat to compensate you for your time for participating in the study and travel and related expenses if you had to travel from your residence.

Sharing the result

Results from this study are expected to be shared nationally and internationally; however, no identifying information will be included with any of the results disseminated. You can request results of the study by contacting the Principal Investigator of the study after April 2018.

Who to contact

If you agree to participate in the study, you can contact the person mentioned below at any time if you have any questions:

Dr Win Han Oo
PhD Candidate
School of Health and Social Development
Faculty of Health, Deakin University
226,4th Floor, U Wisara Road,Wizaya Plaza, Bahan Township 11201, Yangon.
Email: owinhan@deakin.edu.au
Ph: +95-1-375785, 375763, 375727, 512693 Ext 106

For questions regarding study participants' rights, please contact:

Secretary of the Ethics Review Committee
Department of Medical Research
No. 5 Ziwaka Road, Dagon PO Yangon, Myanmar
Phone: 01 375447- ext: 118 during office hours

For complaints please contact:
Complaints Officer
Office of Ethics & Research Governance, Alfred Health
Phone: +61 3 9076 3619, Email: research@alfred.org.au
Note: You will need to quote the following Alfred Health project number: 445/17

PART 2: Certificate of Consent

I have been invited to participate in research about **"Perspectives of community delivered models for the malaria elimination in Myanmar: A qualitative study"**. I understand that it will involve a Focus Group Discussion that will take about one to two hours. I am aware that there may be no benefit to me personally. I am given the contact details of the principal investigator. I have been informed about the rights of the participant.

Written consent:

I have read the information in this consent form. All my questions about the study and my participation in it have been answered. I understand what my involvement in the study means, and I voluntarily agree to participate, and understand that I have the right to withdraw from the study at any time without any consequences.

Name of Participant: ________________________

Signature of Participant: ________________________

Date: ________________________

(Day/month/year)

If illiterate:

A literate **witness must sign** (if possible, this person **should be selected by the participant** and should have **no connection to the research team**). Participants who are illiterate should include their **thumb-print** as well.

Thumb print of participant: ☐

I have witnessed the accurate reading of the consent form to the potential participant, and the individual has had the opportunity to ask questions. I confirm that the individual has given consent freely.

Name of witness: ________________________

Signature of witness: ________________________

Date: ________________________

(Day/month/year)

I have accurately read or witnessed the accurate reading of the consent form to the potential participant, and the individual has had the opportunity to ask questions. I confirm that the individual has given consent freely.

Name of Researcher: ________________________

Signature of Researcher: ________________________

Date: ________________________

(Day/month/year)

A copy of this Informed Consent Form has been provided to the participant _____ (initialled by the researcher/assistant)

လူပုဂ္ဂိုလ်များအပေါ် သုတေသန စမ်းသပ်မှုဆိုင်ရာ ကျင့်ဝတ်ကော်မတီ

ဆေးသုတေသန ဦးစီးဌာန

ကျန်းမာရေး နှင့် အားကစား ဝန်ကြီးဌာန

ပြည်ထောင်စု သမ္မတ မြန်မာနိုင်ငံတော်

(မြန်မာဘာသာပြန်ဆိုချက်)

"မြန်မာနိုင်ငံ ငှက်ဖျားရောဂါ အမြစ်ပြတ်ရှင်းလင်းရေး တွင် **ပြည်သူလူထု အခြေပြု ငှက်ဖျား ကျန်းမာရေးစောင့်ရှောက်မှု ပုံစံများ အပေါ် အမြင်နှင့် ရှုထောင့်များကို ဖော်ထုတ်ခြင်း" ကို အုပ်စုဖွဲ့ဆွေးနွေးခြင်း (အရပ်သားများ)**

ဤသဘောတူခွင့်ပြုလွှာသည် "မြန်မာနိုင်ငံ ငှက်ဖျားရောဂါ အမြ စ်ပြတ်ရှင်းလင်းရေး တွင် **ပြည်သူလူထု အခြေပြု ငှက်ဖျား ကျန်းမာရေးစောင့်ရှောက်မှု ပုံစံများ အပေါ် အမြင်နှင့် ရှုထောင့်များကို ဖော်ထုတ်ခြင်း"** သုတေသနလုပ်ငန်းတွင် ပါဝင်ဆွေးနွေးရန် ဖိတ်ခေါ်ထားသော **အရပ်သားများ** အတွက် ဖြစ်ပါသည်။

အဓိကသုတေသီအမည်	ဒေါက်တာ ဝင်းဟန်ဦး
အဖွဲ့အစည်းအမည်	Deakin တက္ကသိုလ် နှင့် Burnet Institute
ထောက်ပံ့ပေးသောအဖွဲ့အစည်းအမည်	Deakin တက္ကသိုလ် နှင့် Burnet Institute
သုတေသနခေါင်းစဉ်	"မြန်မာနိုင်ငံ ငှက်ဖျားရောဂါ အမြစ်ပြတ်ရှင်းလင်းရေး တွင် ပြည်သူလူထု အခြေပြု ငှက်ဖျား ကျန်းမာရေးစောင့်ရှောက်မှု ပုံစံများ အပေါ် အမြင်နှင့် ရှုထောင့်များကို ဖော်ထုတ်ခြင်း"

အပိုင်း(၁) သုတေသနနှင့်ပတ်သက်သည့်အကြောင်းအရာများ

မိတ်ဆက်ခြင်း

ကျွန်ုပ် --------------------------- သည် မြန်မာနိုင်ငံ Burnet Institute ၏စီမံချက်များ အတွက် အလုပ်လုပ်ကိုင်ပါသည်။ ကျွန်ုပ်သည် "မြန်မာနိုင်ငံ ငှက်ဖျားရောဂါ အမြစ်ပြတ်ရှင်းလင်းရေး တွင် ပြည်သူလူထု အခြေပြု ငှက်ဖျား ကျန်းမာရေးစောင့်ရှောက်မှု ပုံစံများ အပေါ် အမြင်နှင့် ရှုထောင့်များကို ဖော်ထုတ်ခြင်း" သုတေသနအတွက် အုပ်စုဖွဲ့ ဆွေးနွေးခြင်းကို ဦးဆောင် ပြုလုပ်မည် ဖြစ်ပါသည်။ ဤသဘောတူခွင့်ပြုလွှာက သုတေသနအကြောင်း သတင်း အချက်အလက်အချို့ကို ပေးပါမည်။ သဘောတူခွင့်ပြုလွှာကို ဖတ်ပြသွားစဉ် နားမလည်သောစကားရပ်များ (သို့မဟုတ်) အကြောင်းအရာ တို့ကို ပြန်လည်ရှင်းလင်းဖြေကြားပေးနိုင်ရန် ကျွန်ုပ်အား မေးမြန်းနိုင်ပါသည်။ ကျွန်ုပ်တို့သည် လက်မှတ်ရေးထိုးပြီး မိတ္တူ (သို့မဟုတ်) အလိုရှိပါက လက်မှတ် ရေးထိုးမထားသော မိတ္တူကို မှတ်တမ်းအဖြစ် သိမ်းထားနိုင်ရန် ပေးပါမည်။

သုတေသန၏ရည်ရွယ်ချက်

ဤသုတေသနသည် အဓိကသုတေသီ ဒေါက်တာ ဝင်းဟန်ဦး၏ Australia နိုင်ငံ၊ Deakin တက္ကသိုလ် တွင်တက်ရောက်နေသော ပါရဂူဘွဲ့ အတွက် အဓိက ရည်ရွယ်ပြီး၊ Deakin တက္ကသိုလ် နှင့် Burnet Institute တို့မှ ငွေကြေးထောက်ပံ့ပေးပါသည်။ မြန်မာနိုင်ငံသည် မဟာ မဲခေါင်မြစ်ဝှမ်းဒေသနိုင်ငံများအနက် ငှက်ဖျားရောဂါ အဖြစ်အများဆုံး ဖြစ်ပြီး ၂၀၃၀ ခုနှစ်နောက်ဆုံးထား၍

နိုင်ငံတွင်းမှ ငှက်ဖျားရောဂါရှင်းလင်းနိုင်ရန် ရည်ရွယ်ထားပါသည်။ ပါရဂူဘွဲ့ အတွက် စီမံချက်မှာ ယခုလက်ရှိ အသုံးပြုနေသော ပြည်သူလူထု အခြေပြု ငှက်ဖျား ကျန်းမာရေးစောင့်ရှောက်မှု ပုံစံ များနှင့် နှိုင်းယှဉ်ပါက လုပ်ဆောင်မည့်သူများလက်ခံနိုင်လောက်ပြီး၊ လက်တွေ့လုပ်ဆောင်ရာတွင်ဖြစ်နိုင်သော၊ တွေ့ရှိထားသော သက်သေသာကေများကို အခြေခံသည့်၊ ထိရောက်မှုရှိသော ပုံစံသစ်တစ်ခုကို မြန်မာနိုင်ငံ ငှက်ဖျားရောဂါ အမြစ်ပြတ်ရှင်းလင်းရေး အတွက်တည်ဆောက်ရန်ဖြစ်ပါသည်။

ဤသုတေသနသည် မြန်မာနိုင်ငံတွင်းရှိ ကျန်းမာရေးနှင့်စပ်ဆိုင်သော အဓိက ပုဂ္ဂိုလ်များ နှင့် လူထု၏ ပြည်သူလူထုအခြေပြု ငှက်ဖျား ကျန်းမာရေးစောင့်ရှောက်မှု ပုံစံများ အပေါ် အမြင်နှင့် ရှုထောင့်များကို ဖော်ထုတ်ပြီး ပြည်သူလူထုအခြေပြု ငှက်ဖျား ကျန်းမာရေးစောင့်ရှောက်မှု ပုံစံသစ် တစ်ခုကို ငှက်ဖျားရောဂါ အမြစ်ပြတ်ရှင်းလင်းရေး အတွက်တည်ဆောက်ရန်ဖြစ်ပါသည်။

ဤသုတေသနကို အောက်ပါရည်ရွယ်ချက်များဖြင့် ပြုလုပ်ပါသည်

- ယခုလက်ရှိ ငှက်ဖျားရောဂါ တိုက်ဖျက်ရေးတွင် အသုံးပြုနေသော ပြည်သူလူထု အခြေပြု ငှက်ဖျား ကျန်းမာရေးစောင့်ရှောက်မှု ပုံစံ အပေါ် အမြင် နှင့်ရှုထောင့် များကို ဖော်ထုတ်စုစည်းရန်။
- ပြည်သူလူထု အခြေပြု ငှက်ဖျားကျန်းမာရေး စေတ နာ့ဝန်ထမ်းများ၏ ငှက်ဖျားရောဂါ တိုက်ဖျက်နှိမ်နင်းရေး လုပ်ငန်းများ အပေါ် စိတ်အားထက်သန်မှု နှင့် ၎င်းတို ့၏ လူထု အတွင်း အရေးပါမှုကို ထိန်းသိမ်း နိုင်မည့်နည်းလမ်းများကို ဖော်ထုတ်စုစည်းရန်။
- ငှက်ဖျားရောဂါ ထိမ်းချုပ်ရေးမှ ဒေသ တွင်း ငှက်ဖျားရောဂါကင်းဝေးပပျောက်ရေးသို့ အသွင်ကူးပြောင်းရာတွင် ပြည်သူလူထု အခြေပြု ငှက်ဖျားကျန်းမာရေးစောင့်ရှောက်မှု ပုံစံ နှင့် သက်ဆိုင်သော သတိထား ဂရုပြုရမည့် အချက်အလက်များကို ဖော်ထုတ်ရန်။

ကျွန်ုပ်တို့သည် သင်အပါအဝင် ကျန်းမာရေးနှင့် အားကစားဝန်ကြီးဌာနမှ ဝန်ထမ်းများ၊ ငှက်ဖျားဆိုင်ရာ လုပ်ငန်းများ ဆောင်ရွက်နေသည့် အစိုးရမဟုတ်သောအဖွဲ့ အစည်းများမှ ဝန်ထမ်းများ၊ လူထု အတွင်း ဦးဆောင်ဦးရွက်ပြုနေသူများနှင့် လူထုထံမှ သတင်း အချက်အလက်များရယူမည်ဖြစ်ပါသည်။

သုတေသနဆောင်ရွက်ပုံအမျိုးအစား

ဤသုတေသနတွင် သင့်အား သုတေသန၏တစိတ်တပိုင်းဖြစ်သောအဖွဲ့ လိုက်ဆွေးနွေးခြင်းတွင် ပါဝင်ရန်ဖိတ်ခေါ်ပါသည်။ ဤဆွေးနွေးခြင်းတွင်လက်ရှိငှက်ဖျားရောဂါအခြေအနေနှင့်ဦးစားပေးလူထုကျန်းမာရေးပြသနာများ၊ ငှက်ဖျားထိန်းချုပ်ရေးဆောင်ရွက်ချက်များ နှင့်သင်၏ လူ့ အဖွဲ့ အစည်းတွင်ရရှိနိုင်သောကျန်းမာရေးစောင့်ရှောက်မှုလုပ်ငန်းများ၊ လက်ရှိ ပြည်သူလူထု အခြေပြု ငှက်ဖျား ကျန်းမာရေးစောင့်ရှောက်မှု ပုံစံ အပေါ် အမြင် နှင့်ရှုထောင့် များ၊ ငှက်ဖျားရောဂါ ထိန်းချုပ်ရေးနှင့်အမြစ်ပြတ်ရှင်းလင်းရေး အတွက်သင်၏ပတ်ဝန်းကျင်တွင်ရရှိနိုင်သောကူညီထောက်ပံ့မှု များ၊ ပြည်သူလူထု အခြေပြု ငှက်ဖျားကျန်းမာရေး စေတ နာ့ဝန်ထမ်းများ၏ ငှက်ဖျားရောဂါ တိုက်ဖျက်နှိမ်နင်းရေး လုပ်ငန်းများ အပေါ် စိတ်အားထက်သန်မှု နှင့် ၎င်းတို ့၏ လူထု အတွင်း အရေးပါမှုကို ထိန်းသိမ်း နိုင်မည့်နည်းလမ်းများ၊ နှင့်ဓလေ့ထုံးစံ၊လူမှုကျင့်ဝတ်ပိုင်းဆိုင်ရာလက်ခံယုံကြည်မှုတို့ကြောင့် ငှက်ဖျားရောဂါထိန်းချုပ်ရေးနှင့် အမြစ်ပြတ်ရှင်းလင်းရေးလုပ်ငန်းစဉ်များတွင် ဖြစ်ပေါ်လာနိုင်သော အတားအဆီးနှင့် အားသာချက်များစသည့် အကြောင်းအရာများကို မေးမြန်းပါမည်။

သုတေသနတွင် ပါဝင်မည့်သူများကို ရွေးချယ်ခြင်း

သင့်အား ဤသုတေသနတွင် ပါဝင်ရန်ရွေးချယ်ရခြင်းမှာ ငှက်ဖျားဒေသရှိ ပြည်သူတစ်ယောက်အနေဖြင့် သင်၏ အတွေ့အကြုံနှင့် အသိပညာများသည် ဤသုတေသနလုပ်ငန်းအတွက် အလွန်အထောက်အကူပေးနိုင်မည်ဟုယုံကြည်သောကြောင့်ဖြစ်ပါသည်။

မိမိဆန္ဒအလျောက် သုတေသနတွင်ပါဝင်ခြင်း

သင်၏ ပါဝင်မှုသည် သင်၏ လွတ်လပ်သော ဆန္ဒအလျောက် ဖြစ်ပါသည်။ ဤသုတေသနလုပ်ငန်းတွင် ပါဝင်ခြင်းပြု၊ မပြု (သို့ မဟုတ်) အချိန်မရွေးနှုတ်ထွက်ခြင်းတို့သည် သင်၏အခွင့်အရေး ရပိုင်ခွင့်ဖြစ်ပါသည်။ သင်မဆွေးနွေးလိုသောအကြောင်းအရာများပါဝင်ပါက မဖြေဘဲနေနိုင်ပါသည်။ ဤသုတေသနတွင်ပါဝင်ခြင်း၊ မပါဝင်ခြင်း နှင့် သင်ဖြေကြားသောအကြောင်းအရာများသည် သင်၏ကျန်းမာရေးစောင့်ရှောက်မှုကို မည်သို့မျှထိခိုက်စေမည် မဟုတ်ပါ။

သုတေသနလုပ်ငန်းလုပ်ဆောင်ချက်အဆင့်ဆင့်

အဖွဲ့လိုက်ဆွေးနွေးခြင်းတွင် အရပ်သား ၆ ယောက် မှ ၈ယောက်ပါဝင်မည်ဖြစ်ပြီး ဦးဆောင်ဆွေးနွေးသူတစ်ဦး နှင့်ဆွေးနွေးချက်များကို ရေးခြစ်မှတ်သားမည့်သူတစ်ဦးတို့က ပြုလုပ်ပါမည်။ ဆွေးနွေးခြင်းကို လုံခြုံမှုရှိပြီး အခြားသူများမကြားနိုင်သော နေရာတွင်ပြုလုပ်ပါမည်။ ခေါင်းစဉ်များကိုဆွေးနွေးရာတွင် အမျိုးသားဆိုလျှင် အမျိုးသားအုပ်စု၊ အမျိုးသမီးဆိုလျှင်အမျိုးသမီးအုပ်စုအလိုက် ဆွေးနွေးမည်ဖြစ်သည်။ ဆွေးနွေးချက်များကို စာရွက်ပေါ်တွင် ရေးခြစ်မှတ်သားပြီး အသံလည်းသွင်းယူထားမည်ဖြစ်ပါသည်။ သို့သော် သင်၏အမည်အား စာရွက်ပေါ်တွင်ဇင်း၊ အသံသွင်းရာတွင်ဇင်း မှတ်သားမည် မဟုတ်ပါ။

ဆွေးနွေးပွဲ မစမီတွင် လူမှုအဖွဲ့အတွင်းရှိ မိမိ၏ တာဝန်ယူတာဝန်ခံမှုအပိုင်း၊ အသက်၊ ကျား/မ၊ အလုပ်အကိုင် အစရှိသည့်တစ်ဦး တစ်ယောက်ကို ဖော်ထုတ်သတ်မှတ်နိုင်ခြင်းမပြုသည့် အချက်အလက်များကို ဦးဆောင်ဆွေးနွေးသူမှမေးမြန်း ရယူမည်ဖြစ်သည်။ ဤဆွေးနွေးခြင်းတွင် လက်ရှိငှက်ဖျားရောဂါအခြေအနေနှင့် ဦးစားပေးလူထုကျန်းမာရေးပြဿနာများ၊ ငှက်ဖျားထိန်းချုပ်ရေးဆောင်ရွက်ချက်များ နှင့်သင်၏ လူ့အဖွဲ့အစည်းတွင်ရရှိနိုင်သောကျန်းမာရေးစောင့်ရှောက်မှုလုပ်ငန်းများ၊ လက်ရှိ ပြည်သူလူထု အခြေပြု ငှက်ဖျား ကျန်းမာရေးစောင့်ရှောက်မှု ပုံစံ အပေါ် အမြင် နှင့်ရှုထောင့် များ၊ ငှက်ဖျားရောဂါ ထိန်းချုပ်ရေးနှင့်အမြစ်ပြတ်ရှင်းလင်းရေး အတွက်သင်၏ပတ်ဝန်းကျင်တွင်ရရှိနိုင်သောကူညီထောက်ပံ့မှုများ၊ ပြည်သူလူထု အခြေပြု ငှက်ဖျားကျန်းမာရေး စေတနာ့ဝန်ထမ်းများ၏ ငှက်ဖျားရောဂါ တိုက်ဖျက်နှိမ်နင်းရေး လုပ်ငန်းများ အပေါ် စိတ်အားထက်သန်မှု နှင့် ၎င်းတို့၏ လူထု အတွင်း အရေးပါမှုကို ထိန်းသိမ်း နိုင်မည့်နည်းလမ်းများနှင့် ဓလေ့ထုံးစံ၊လူမှုကျင့်ဝတ်ပိုင်းဆိုင်ရာ လက်ခံယုံကြည်မှုတို့ကြောင့် ငှက်ဖျားရောဂါထိန်းချုပ်ရေးနှင့် အမြစ်ပြတ်ရှင်းလင်းရေးလုပ်ငန်းစဉ်များတွင်ဖြစ်ပေါ်လာနိုင်သော အတားအဆီးနှင့် အားသာချက်များစသည့် အကြောင်းအရာများကို ဆွေးနွေးမေးမြန်းပါမည်။

အချိန်ကြာမြင့်မှု

ဆွေးနွေးပွဲသည် ခန့်မှန်းခြေ တစ်နာရီမှ နှစ်နာရီခန့် ကြာမြင့်မည် ဖြစ်ပါသည်။

အချက်အလက်များကို လျှို့ပုက်ထားရှိမှု

ဤသုတေသနမှ ရရှိသော သတင်းအချက်အလက်များကို လျှို့ပုက်ထားရှိပါမည်။ သုတေသနအဖွဲ့ဝင်မှလွဲ၍ မည်သူတစ်ဦးတစ်ယောက်မျှ ကြည့်ရှုနားဆင်ပိုင်ခွင့် မရှိပါ။ ဤသုတေသနကို ဆောင်ရွက်သူများသာလျှင် သင်ပြောပြသော အချက်အလက်များကို ရရှိနိုင်မည် ဖြစ်သည်။ သင်၏ အမည် (သို့မဟုတ်) အခြား သင့်ကို မှတ်သားဖေါ်ထုတ်နိုင်သော အချက်အလက်များကို မှတ်သားဖေါ်ပြထားမည် မဟုတ်ပါ။ သင်ပါဝင်ရန် သဘောတူပါက သင့်အတွက် အမည်လွှဲတစ်ခုဖြင့် သတ်မှတ်ထားပြီး သုတေသနလုပ်ငန်းစဉ်တစ်လျှောက် သင်၏ လွတ်လပ်မှုကို ကာကွယ်ပေးထားမည်ဖြစ်သည်။

ဤမှတ်စုများ နှင့် အသံသွင်းထားသော ကိရိယာများ အားလုံးကို ဘီရိုထဲတွင် သော့ခတ်ပြီးသိမ်းဆည်းထားမည်ဖြစ်၍ သုတေသနဆောင်ရွက်သူများကသာ ရယူနိုင်ပါမည်။ ထို့အတူ ဤသဘောတူခွင့်ပြုလွှာတွင် လက်မှတ်ရေးထိုးခြင်းဖြင့် The Alfred Office of Ethics & Research Governance ၏ ဆန်းစစ်မှု ဘုတ်အဖွဲ့နှင့် ဆေးသုတေသနဦးစီးဌာန လူပုဂ္ဂိုလ်များအပေါ် သုတေသန စမ်းသပ်မှုဆိုင်ရာ ကျင့်ဝတ်ကော်မီတီတို့၏ ကိုယ်စားလှယ်များကလေ့လာမှု၏ မှတ်တမ်းများကို ကြည့်ရှုနိုင်ရန် သဘောတူခွင့်ပြုချက် ပေးခြင်းဖြစ်သည်။ သို့သော် ထိုအဖွဲ့များက သင်၏ ကိုယ်ပိုင်လွတ်လပ်ခွင့်ကို ထိန်းသိမ်းကာကွယ်ပေးထားကြောင်း သေချာစေရန်အလို့ငှာသာ ကြည့်ရှု စစ်ဆေးမည်ဖြစ်ပါသည်။ အသံ မှတ်တမ်း များကို နားထောင်ပြီး ဆွေးနွေးပြောဆိုမှုများကို ရေးသားမှတ်တမ်းတင်မည် ဖြစ်ပါသည်။ အသုံးပြုထားသော အသံသွင်းစက်အတွင်းရှိ အသံ မှတ်တမ်း များကို လျှို့ပုက် ဂဏန်းဖြင့် ကာကွယ်ထားသော ကွန်ပျူတာထဲသို့ ကူးယူပြီးနောက် ဖျက်ဆီးပစ်မည်ဖြစ်ပါသည်။ ရေးသားထားသော မှတ်တမ်းများနှင့် အခြားမှတ်သားချက်များ အားလုံးအား ၇ နှစ် အထိ လုံခြုံစွာ သိမ်းဆည်းထားမည် ဖြစ်ပြီး၊ ၇ နှစ်ကျော်ပါက ဖျက်ဆီး ပစ်မည်ဖြစ်ပါသည်။

ထိခိုက်နိုင်မှုနှင့် ကိုယ်စိတ် အနှောင့်အယှက်ဖြစ်စေခြင်းများ

ဤဆွေးနွေးခြင်းတွင် လက်ရှိငှက်ဖျားရောဂါအခြေအနေနှင့် ဦးစားပေးလူထုကျန်းမာရေးပြဿနာများ ၊ငှက်ဖျားထိန်းချုပ်ရေးဆောင်ရွက်ချက်များ နှင့်သင်၏ လူ့ အဖွဲ့ အစည်းတွင်ရရှိနိုင်သောကျန်းမာရေးစောင့်ရှောက်မှုလုပ်ငန်းများ၊ လက်ရှိ ပြည်သူလူထု အခြေပြု ငှက်ဖျား ကျန်းမာရေးစောင့်ရှောက်မှု ပုံစံ အပေါ် အမြင် နှင့်ရှုထောင့် များ၊ ငှက်ဖျားရောဂါ ထိန်းချုပ်ရေးနှင့်အမြစ်ပြတ်ရှင်းလင်းရေး အတွက်သင်၏ပတ်ဝန်းကျင်တွင်ရရှိနိုင်သောကူညီထောက်ပံ့မှု များ၊ ပြည်သူလူထု အခြေပြု ငှက်ဖျားကျန်းမာရေး စေတနာ့ဝန်ထမ်းများ၏ ငှက်ဖျားရောဂါ တိုက်ဖျက်နှိမ်နင်းရေး လုပ်ငန်းများ အပေါ် စိတ်အားထက်သန်မှု နှင့် ၎င်းတို့၏ လူထု အတွင်း အရေးပါမှုကို ထိန်းသိမ်း နိုင်မည့်နည်းလမ်းများ၊ နှင့်ဓလေ့ထုံးစံ၊လူမှုကျင့်ဝတ်ပိုင်းဆိုင်ရာ လက်ခံယုံကြည်မှုတို့ကြောင့် ငှက်ဖျားရောဂါထိန်းချုပ်ရေးနှင့် အမြစ်ပြတ်ရှင်းလင်းရေးလုပ်ငန်းစဉ်များတွင် ဖြစ်ပေါ်လာနိုင်သော အတားအဆီးနှင့်အားသာချက်များစသည့် အကြောင်းအရာများကိုမေးမြန်းပါမည်။ မေးခွန်းများကိုဖြေကြားရာ၌ အနည်းငယ် စိတ်မသက်မသာဖြစ်နိုင်ပါသည်။ သို့ရာတွင် သင့်အား စိတ်အနှောင့် အယှက်ဖြစ်စေမည့် အကြောင်းအရာများ ပါဝင်မှုမရှိစေရန် မျှော်လင့်ပါသည်။ အချို့သောအကြောင်းအရာများသည် သင့်အား စိတ်အနှောင့် အယှက်ဖြစ်စေမည်ဆိုလျှင် မဖြေဘဲ၊ မဆွေးနွေးဘဲ ထားနိုင်ပါသည်။

အကျိုးကျေးဇူးများ

ဤသုတေသနတွင်ပါဝင်၍ သင့်အတွက် တိုက်ရိုက်အကျိုးကျေးဇူးမရှိပါ။ သို့သော် သင်ပါဝင် ဖြေကြားပေးသည့်အတွက် အဓိက သုတေသီ ဒေါက်တာဝင်းဟန်ဦး ၏ ပြည်သူလူထု အခြေပြု ငှက်ဖျား ကျန်းမာရေးစောင့်ရှောက်မှု ပုံစံ အား သုတေသနပြုတည်ဆောက်ရာတွင် ကူညီပေးမည် ဖြစ်ပါသည်။

ကျေးဇူးတုံ့ပြန်မှု

သုတေသနတွင် ပါဝင်မှုအတွက် အစားအသောက်၊ သင်၏အချိန်ပေးရမှုအတွက် မြန်မာကျပ်ငွေ ၄၀၀၀ ကျပ်တိတိ နှင့် အမှန်တကယ်ကုန်ကျသော ခရီးစားရိတ်တို့ကို ပြန်လည်ထောက်ပံ့မည်ဖြစ်ပါသည်။

သုတေသန၏ ရလဒ်များကို ဖြန့်ဝေခြင်း

ဤသုတေသနရလဒ်များကို ပြည်တွင်းနှင့် ပြည်ပတွင် သုတေသနစာတမ်းအနေဖြင့် ဖြန့်ဝေရန် မျှော်လင့်ပါသည်။ သို့သော်လည်း တစ်ဦးတစ်ယောက်ကို ဖော်ထုတ်သတ်မှတ်နိုင်သည့်သတင်းအချက်အလက်ပါဝင်မည်မဟုတ်ပါ။ သုတေသနရလဒ်များကို အဓိကသုတေသီထံတွင် ၂၀၁၈ခုနှစ် ဧပြီလနောက်ပိုင်းတွင် တောင်းယူနိုင်ပါသည်။

ဆက်လက်မပါဝင်လိုကြောင်း ငြင်းဆန်နိုင်ခွင့်

သင့်အနေနှင့် သုတေသနတွင် ဆက်လက်မပါဝင်လိုတော့လျှင် အချိန်မရွေး ငြင်းဆန်ပိုင်ခွင့်ရှိပြီး ထိုငြင်းဆန်မှုသည် သင်၏ မူလအခွင့်အရေးများကို ထိခိုက်စေမည် မဟုတ်ပါ။

ဆက်သွယ်နိုင်မည့်ပုဂ္ဂိုလ်များ

သုတေသနတွင်ပါဝင်ဆောင်ရွက်ရန်သဘောတူပါက အောက်ပါပုဂ္ဂိုလ်ထံ အချိန်မရွေးဆက်သွယ်မေးမြန်းနိုင်ပါသည်။

ဒေါက်တာ ဝင်းဟန်ဦး
ပါရဂူ ကျောင်းသား
ကျန်းမာရေးဆိုင်ရာ ဌာန၊ Deakin တက္ကသိုလ်
၂၂၆၊ ၄ လွှာ၊ ဦးဝိစာရလမ်း၊ ဝိဇယပလာဇာ၊ ဗဟန်းမြို့နယ်၊ ရန်ကုန်မြို့။
ဖုန်း +95-1-375785, 375763, 375727, 512693 Ext 106
Email: owinhan@deakin.edu.au

ပါဝင်ဆောင်ရွက်သူများ၏ အခွင့်အရေးများနှင့် ပတ်သက်၍ မေးစရာများရှိပါက အောက်ပါ ပုဂ္ဂိုလ်များထံ ဆက်သွယ်နိုင်ပါသည်။

အတွင်းရေးမှူး
လူပုဂ္ဂိုလ်များအပေါ် သုတေသနစမ်းသပ်မှုဆိုင်ရာ ကျင့်ဝတ်ကော်မတီ
ဆေးသုတေသနဦးစီးဌာန၊
အမှတ် ၅ ဇီဝကလမ်း၊ ဒဂုံစာတိုက်၊ ၁၁၁၉၁၊ ရန်ကုန်၊ မြန်မာနိုင်ငံ၊
ဖုန်း ဝ၁ ၃၇၅၄၄၇ လိုင်းခွဲ ၁၁၈ (ရုံးချိန်အတွင်း)

Complaints Officer
Office of Ethics & Research Governance, Alfred Health
Phone: +61 3 9076 3619, Email: research@alfred.org.au

Note: You will need to quote the following Alfred Health project number: 445/17

အပိုင်း၂၊ သဘောတူညီချက်

ကျွန်ုပ်သည် "မြန်မာနိုင်ငံ ငှက်ဖျားရောဂါ အမြစ်ပြတ်ရှင်းလင်းရေး တွင် **ပြည်သူလူထု အခြေပြု ငှက်ဖျား ကျန်းမာရေးစောင့်ရှောက်မှု ပုံစံများ အပေါ် အမြင်နှင့် ရှုထောင့်များကို ဖော်ထုတ်ခြင်း"** လုပ်ငန်းတွင် ပါဝင်ရန် ဖိတ်ခေါ်ခြင်းခံရပါသည်။ ဤသုတေသနတွင် အချိန် တစ်နာရီမှ နှစ်နာရီခန့် ကြာမြင့်သောဆွေးနွေးခြင်းပါဝင်မည်ဖြစ်ကြောင်း ကျွန်ုပ်အနေဖြင့် သိရှိပြီးဖြစ်ပါသည်။ ကျွန်ုပ်အတွက် ကိုယ်ရေးကိုယ်တာ အကျိုးအမြတ် ရရှိမည် မဟုတ်ကြောင်းကိုလည်း သိရှိပြီးဖြစ်ပါသည်။ သုတေသနဆောင်ရွက်သူနှင့် ဆက်သွယ်ရန် လိပ်စာနှင့် ဖုန်းနံပါတ်များကိုလည်း သိရှိပြီးဖြစ်ပါသည်။ ကျွန်ုပ်သည် ရှေ့မှအချက်အလက်များကို ဖတ်ရှုပြီးဖြစ်ပါသည် (သို့မဟုတ်) ကျွန်ုပ်အား ဖတ်ပြပြီးဖြစ်ပါသည်။ ကျွန်ုပ်တွင် မေးခွန်းမေးပိုင်ခွင့်နှင့် ထိုမေးခွန်းများကို ကျွန်ုပ်ကျေနပ်သည်အထိ ဖြေကြားပြီး ဖြစ်ပါသ ည်။ ကျွန်ုပ်သည် သုတေသနတွင် မိမိဆန္ဒ အလျောက်ပါဝင်ရန် သဘောတူပါသည်။ ဤသုတေသနလုပ်ငန်းမှ အချိန်မရွေး နုတ်ထွက်ခွင့်ရှိပြီး၊ ယင်းသို့ နုတ်ထွက်ခြင်းကြောင့် ကျွန်ုပ်၏ ကျန်းမာရေးစောင့်ရှောက်မှု ရပိုင်ခွင့်များကို ထိခိုက်မှုမရှိကြောင်း နားလည်ပြီးဖြစ်သည်။

ပါဝင်သူအမည် ------------------------

ပါဝင်သူလက်မှတ် ---------------------

ရက်စွဲ၊ --------------------------------

ရက် လ နှစ်

ပါဝင်သူသည် စာမတတ်ပါက စာတတ်သော သက်သေတစ်ဦးမှ လက်မှတ်ရေးထိုးရမည် (ဖြစ်နိုင်ပါက ထိုသက်သေကို ပါဝင်သူမှရွေးချယ်ရမည်ဖြစ်ပြီး သုတေသန အဖွဲ့နှင့် ဆက်စပ်မှု မရှိရပါ)။ စာမတတ်သောပါဝင်သူသည် အောက်တွင်လက်ဗွေနှိပ်ရမည်ဖြစ်သည်။

လက်ဗွေနှိပ်ရန်

ကျွန်ုပ်သည် မေးမြန်းသူကပါဝင်သူအား သဘောတူခွင့်ပြုလွှာကို တိကျသေချာစွာဖတ်ပြခြင်းကို တွေ့ရှိပြီးဖြစ်ပါသည်။ ပါဝင်သူမှလဲ မေးခွန်းများပြန်လည် မေးမြန်းခွင့်ရှိခဲ့ပါသည်။ ပါဝင်ဖြေဆိုသူသည် လွတ်လပ်စွာ မိမိသဘောဆန္ဒအလျောက် ဖြေဆို ခြင်းဖြစ်ကြောင်း အတည်ပြုပါသည်။

သက်သေအမည် ------------------------

သက်သေ၏လက်မှတ် ---------------------

ရက်စွဲ၊ --------------------------------

ရက် လ နှစ်

ကျွန်ုပ်သည် ပါဝင်ဖြေဆိုရန် အလားအလာရှိသူအား သဘောတူခွင့်ပြုလွှာကို သေချာစွာဖတ်ပြခဲ့ပါသည်။ (သို့မဟုတ်) ဖတ်ပြသည်ကို တွေ့ရှိ ပါသည်။ ပြီးနောက် မေးခွန်းများ ပြန်လှန်မေးမြန်းခွင့် ပေးခဲ့ပါသည်။ ပါဝင်ဖြေဆိုသူသည် လွတ်လပ်စွာ မိမိသဘောဆန္ဒအလျောက် ဖြေဆို ခြင်းဖြစ်ကြောင်း အတည်ပြုပါသည်။

တွေ့ဆုံမေးမြန်းသူအမည် ------------------------

တွေ့ဆုံမေးမြန်းသူ လက်မှတ် ---------------------

ရက်စွဲ၊ --------------------------------

ရက် လ နှစ်

ဤသဘောတူခွင့်ပြုလွှာ မိတ္တူတစ်စောင်ကို သုတေသနတွင် ပါဝင်မည့်သူအား ပေးအပ်ပြီး ဖြစ်ပါသည်။--------------------------- (သုတေသီ၏ လက်မှတ်တို)

Ethics Review Committee
Department of Medical Research
Ministry of Health and Sports
Republic of the Union of Myanmar

Informed Consent Form for "Perspectives of community delivered models for the malaria elimination in Myanmar: A qualitative study", participatory workshop with community leaders

This informed consent form is for **community leaders**, invited to participate in the **"Perspectives of community delivered models for the malaria elimination in Myanmar: A qualitative study".**

**Name of Principal Investigator:**	Dr. Win Han Oo
**Name of Organization:**	Deakin University and Burnet Institute
**Name of sponsor:**	Deakin University and Burnet Institute
**Title of study:**	"Perspectives of community delivered models for the malaria elimination in Myanmar: A qualitative study"

PART 1: Information Sheet

Introduction

I am ______________ and I work for the Burnet Institute funded project in Myanmar. I am conducting a study on "Perspectives of community delivered models for the malaria elimination in Myanmar: A qualitative study". This consent form will provide you with some information about the study. It may contain words that you do not understand. Please ask me to explain any words or information that you do not clearly understand as we go through the form. I will also give you a signed copy (or unsigned, if you wish) to keep for your record.

Purpose of the research

This study is being conducted by Dr Win Han Oo for his PhD degree at Deakin University Australia, and is funded by Deakin University and Burnet Institute. Myanmar has the highest burden of malaria in the Greater Mekong Sub-region and aims to eliminate malaria by 2030. The PhD project aims to develop a community-delivered malaria elimination model that is acceptable, operational, pragmatic, evidence based and effective compared to the traditional model for malaria elimination in Myanmar.

This study aims to explore the perspectives, views and inputs of key health stakeholders and community members in Myanmar on community-delivered models for the development of community- delivered malaria elimination model. We are doing research to:

- explore the views and perspectives on the current malaria CHW models
- explore the strategies to maintain the motivation and social role of CHWs in the community
- explore the factors that need to be addressed during the transition from malaria control to elimination setting focusing on the community delivered models

We are collecting information from health staff, malaria implementing partner staff, community leaders and community members.

Type of research intervention

You are invited to participate in a participatory workshop as part of this study. In this workshop, we will work on and brain storm together about the current malaria situation and priority health problems, malaria control measures and available health services in your community, your views and perspectives on the current malaria Community Health Worker (CHW) models, policy and strategic barriers and enablers for effective malaria control and elimination in your community, available community supports for malaria control and elimination in the community, strategies to maintain the motivation and social role of CHWs in the community and culture, customs and norms of the ethnic communities that act as barriers and enablers for effective malaria control and elimination in your community.

Participant selection

You are being invited to take part in this research because we feel that your experience and knowledge as a community leader can contribute much to our understanding and knowledge of community delivered models for malaria elimination in Myanmar.

Voluntary Participation

Your participation is voluntary. It is your right to decide whether or not you want to join the study or to stop participating at any time. You are not required to answer any questions or participate in any activity that you are not comfortable with. Whether you choose to participate in this study or not, and any information you choose to provide to us, will not affect the healthcare you receive in any way.

Procedure

The workshop will be led by the PI Win Han Oo and at least two in-country research team members. The participants will be community leaders like you representing the respective communities. The discussions in the workshop will be audio recorded and field notes will be taken after you have given informed consent. The workshop will be conducted in a secure location such as a church or a community leader's house which can guarantee privacy.

The topics to be covered in the workshop are: (i) introduction to malaria and situation of malaria in their villages; (ii) current malaria interventions in their communities; (iii) current malaria service delivery model in the community; (iv) transition from malaria control to elimination and impact on the community; (v) brainstorming; (vi) discussion and group consensus for each theme; (vii) conclusion and group recommendations from the workshop. You will be grouped according to your backgrounds for the brainstorming sessions and Participatory Learning Action (PLA) tools will be used. Prior to commencing the workshop, the facilitator will obtain non-identifying information relating to the role/responsibility in the community, age and residing township.

Duration
The duration of the workshop will be approximately one day (9 am – 5 pm).

Confidentiality
Information obtained in this study will be kept confidential and will not be shared with anyone outside the study team. Your name or other identifying information will not appear in the notes from this discussion or in the final report, and only staff participating in the study will have access to the information you provide. Once you agree to participate in the study, we will ask you to choose a pseudonym (a fake name) in order to try to protect your privacy throughout your participation.

All consent forms, audio recordings, and notes from this study will be stored in a locked filing cabinet, and only study staff will have access to them. Representatives of "The Alfred Office of Ethics & Research Governance" and "Department of Medical Research Ethics Review Committee" will be able to access these documents. However they would only do this to ensure that your privacy is being maintained and protected. We will use the audio recordings to produce a written record of our conversation. The audio recordings will then be destroyed after they have been copied to a password protected computer. The written record and any notes from this discussion will be stored securely for a period of seven years and then destroyed.

Risk and Discomforts
During the course of the workshop, you will be asked to brainstorm, investigate and talk about malaria elimination in your community. . There is a small risk that you may feel uncomfortable answering these questions and participating in the PLA activities. However, we do not wish this to happen, and you may refuse to answer any question or not take part in the study if you feel uncomfortable answering any question(s) or participating in any activity(s).

Benefits
You will not get any direct benefit from participating in this study; however, your participation in this study will help the principal investigator Dr Win Han Oo create the community-delivered

malaria elimination model that is acceptable, operational, pragmatic, evidence based and effective compared to the traditional model for malaria elimination in Myanmar.

Incentives
We will provide refreshment, 10000 Kyat to compensate you for your time for participating in the study and travel and related expenses if you had to travel from your residence.

Sharing the result
Results from this study are expected to be shared nationally and internationally; however, no identifying information will be included with any of the results disseminated. You can request results of the study by contacting the Principal Investigator of the study after April 2018.

Who to contact
If you agree to participate in the study, you can contact the person mentioned below at any time if you have any questions:

Dr Win Han Oo
PhD Candidate
School of Health and Social Development
Faculty of Health, Deakin University
226,4th Floor, U Wisara Road,Wizaya Plaza, Bahan Township 11201, Yangon.
Email: owinhan@deakin.edu.au
Ph: +95-1-375785, 375763, 375727, 512693 Ext 106

For questions regarding study participants' rights, please contact:

Secretary of the Ethics Review Committee
Department of Medical Research
No. 5 Ziwaka Road, Dagon PO Yangon, Myanmar
Phone: 01 375447- ext: 118 during office hours

For complaints please contact:
Complaints Officer
Office of Ethics & Research Governance, Alfred Health
Phone: +61 3 9076 3619, Email: research@alfred.org.au
Note: You will need to quote the following Alfred Health project number: 445/17

PART 2: Certificate of Consent

I have been invited to participate in research about **"Perspectives of community delivered models for the malaria elimination in Myanmar: A qualitative study"**. I understand that it will involve a participatory workshop that will take about eight hours. I am aware that there may be no benefit to me personally. I am given the contact details of the principal investigator. I have been informed about the rights of the participant.

Written consent:

I have read the information in this consent form. All my questions about the study and my participation in it have been answered. I understand what my involvement in the study means, and I voluntarily agree to participate, and understand that I have the right to withdraw from the study at any time without any consequences.

Name of Participant: ________________________

Signature of Participant: ________________________

Date: ________________________

(Day/month/year)

If illiterate:

A literate **witness must sign** (if possible, this person **should be selected by the participant** and should have **no connection to the research team**). Participants who are illiterate should include their **thumb-print** as well.

Thumb print of participant: ☐

I have witnessed the accurate reading of the consent form to the potential participant, and the individual has had the opportunity to ask questions. I confirm that the individual has given consent freely.

Name of witness: ________________________

Signature of witness: ________________________

Date: ________________________

(Day/month/year)

I have accurately read or witnessed the accurate reading of the consent form to the potential participant, and the individual has had the opportunity to ask questions. I confirm that the individual has given consent freely.

Name of Researcher: ________________________

Signature of Researcher: ________________________

Date: ________________________

(Day/month/year)

A copy of this Informed Consent Form has been provided to the participant _____ (initialled by the researcher/assistant)

လူပုဂ္ဂိုလ်များအပေါ် သုတေသန စမ်းသပ်မှုဆိုင်ရာ ကျင့်ဝတ်ကော်မတီ

ဆေးသုတေသန ဦးစီးဌာန

ကျန်းမာရေး နှင့် အားကစား ဝန်ကြီးဌာန

ပြည်ထောင်စု သမ္မတ မြန်မာနိုင်ငံတော်

(မြန်မာဘာသာပြန်ဆိုချက်)

"မြန်မာနိုင်ငံ ငှက်ဖျားရောဂါ အမြစ်ပြတ်ရှင်းလင်းရေး တွင် ပြည်သူလူထု အခြေပြု ငှက်ဖျား ကျန်းမာရေးစောင့်ရှောက်မှု ပုံစံများ အပေါ် အမြင်နှင့် ရှုထောင့်များကို ဖော်ထုတ်ခြင်း" ကို ပူးပေါင်းပါဝင်အလုပ်ရုံဆွေးနွေးခြင်း (အရပ်ဖက်ခေါင်းဆောင်များ)

ဤသဘောတူခွင့်ပြုလွှာသည် "မြန်မာနိုင်ငံ ငှက်ဖျားရောဂါ အမြစ်ပြတ်ရှင်းလင်းရေး တွင် **ပြည်သူလူထု အခြေပြု ငှက်ဖျား ကျန်းမာရေးစောင့်ရှောက်မှု ပုံစံများ အပေါ် အမြင်နှင့် ရှုထောင့်များကို ဖော်ထုတ်ခြင်း"** သုတေသနလုပ်ငန်းတွင် ပါဝင်ဆွေးနွေးရန် ဖိတ်ခေါ်ထားသော **အရပ်ဖက်ခေါင်းဆောင်များ** အတွက် ဖြစ်ပါသည်။

အဓိကသုတေသီအမည်	ဒေါက်တာ ဝင်းဟန်ဦး
အဖွဲ့အစည်းအမည်	Deakin တက္ကသိုလ် နှင့် Burnet Institute
ထောက်ပံ့ပေးသောအဖွဲ့အစည်းအမည်	Deakin တက္ကသိုလ် နှင့် Burnet Institute
သုတေသနခေါင်းစဉ်	"မြန်မာနိုင်ငံ ငှက်ဖျားရောဂါ အမြစ်ပြတ်ရှင်းလင်းရေး တွင် ပြည်သူလူထု အခြေပြု ငှက်ဖျား ကျန်းမာရေးစောင့်ရှောက်မှု ပုံစံများ အပေါ် အမြင်နှင့် ရှုထောင့်များကို ဖော်ထုတ်ခြင်း"

အပိုင်း(၁) သုတေသနနှင့်ပတ်သက်သည့်အကြောင်းအရာများ

မိတ်ဆက်ခြင်း

ကျွန်ုပ် --------------------------- သည် မြန်မာနိုင်ငံ Burnet Institute ၏စီမံချက်များ အတွက် အလုပ်လုပ်ကိုင်ပါသည်။ ကျွန်ုပ်သည် "မြန်မာနိုင်ငံ ငှက်ဖျားရောဂါ အမြစ်ပြတ်ရှင်းလင်းရေး တွင် ပြည်သူလူထု အခြေပြု ငှက်ဖျား ကျန်းမာရေးစောင့်ရှောက်မှု ပုံစံများ အပေါ် အမြင်နှင့် ရှုထောင့်များကို ဖော်ထုတ်ခြင်း" သုတေသနအတွက် အလုပ်ရုံဆွေးနွေးပွဲတွင် ဦးဆောင်ပါဝင်မည့်သူတစ်ဦး ဖြစ်ပါသည်။ ဤသဘောတူခွင့်ပြုလွှာက သုတေသနအကြောင်း သတင်း အချက်အလက်အချို့ကို ပေးပါမည်။ သဘောတူခွင့်ပြုလွှာကို ဖတ်ပြသွားစဉ် နားမလည်သောစကားရပ်များ (သို့မဟုတ်) အကြောင်းအရာ တို့ကို ပြန်လည်ရှင်းလင်းဖြေကြားပေးနိုင်ရန် ကျွန်ုပ်အား မေးမြန်းနိုင်ပါသည်။ ကျွန်ုပ်တို့သည် လက်မှတ်ရေးထိုးပြီး မိတ္တူ (သို့မဟုတ်) အလိုရှိပါက လက်မှတ် ရေးထိုးမထားသော မိတ္တူကို မှတ်တမ်းအဖြစ် သိမ်းထားနိုင်ရန် ပေးပါမည်။

သုတေသန၏ရည်ရွယ်ချက်

ဤသုတေသနသည် အဓိကသုတေသီ ဒေါက်တာ ဝင်းဟန်ဦး၏ Australia နိုင်ငံ၊ Deakin တက္ကသိုလ် တွင်တက်ရောက်နေသော ပါရဂူဘွဲ့ အတွက် အဓိက ရည်ရွယ်ပြီး၊ Deakin တက္ကသိုလ် နှင့် Burnet Institute တို့မှ ငွေကြေးထောက်ပံ့ပေးပါသည်။ မြန်မာနိုင်ငံသည် မဟာ မဲခေါင်မြစ်ဝှမ်းဒေသနိုင်ငံများအနက် ငှက်ဖျားရောဂါ အဖြစ်အများဆုံး ဖြစ်ပြီး ၂၀၃၀ ခုနှစ်နောက်ဆုံးထား၍

နိုင်ငံတွင်းမှ ငှက်ဖျားရောဂါရှင်းလင်းနိုင်ရန် ရည်ရွယ်ထားပါသည်။ ပါရဂူဘွဲ့ အတွက် စီမံချက်မှာ ယခုလက်ရှိ အသုံးပြုနေသော ပြည်သူလူထု အခြေပြု ငှက်ဖျား ကျန်းမာရေးစောင့်ရှောက်မှု ပုံစံ များနှင့် နှိုင်းယှဉ်ပါက လုပ်ဆောင်မည့်သူများလက်ခံနိုင်လောက်ပြီး၊ လက်တွေ့လုပ်ဆောင်ရာတွင်ဖြစ်နိုင်သော၊ တွေ့ရှိထားသော သက်သေသာဓကများကို အခြေခံသည့်၊ ထိရောက်မှုရှိသော ပုံစံသစ်တစ်ခုကို မြန်မာနိုင်ငံ ငှက်ဖျားရောဂါ အမြစ်ပြတ်ရှင်းလင်းရေး အတွက်တည်ဆောက်ရန်ဖြစ်ပါသည်။

ဤသုတေသနသည် မြန်မာနိုင်ငံတွင်းရှိ ကျန်းမာရေးနှင့်စပ်ဆိုင်သော အဓိက ပုဂ္ဂိုလ်များ နှင့် လူထု၏ ပြည်သူလူထုအခြေပြု ငှက်ဖျား ကျန်းမာရေးစောင့်ရှောက်မှု ပုံစံများ အပေါ် အမြင်နှင့် ရှုထောင့်များကို ဖော်ထုတ်ပြီး ပြည်သူလူထုအခြေပြု ငှက်ဖျား ကျန်းမာရေးစောင့်ရှောက်မှု ပုံစံသစ် တစ်ခုကို ငှက်ဖျားရောဂါ အမြစ်ပြတ်ရှင်းလင်းရေး အတွက်တည်ဆောက်ရန်ဖြစ်ပါသည်။
ဤသုတေသနကို အောက်ပါရည်ရွယ်ချက်များဖြင့် ပြုလုပ်ပါသည်

- ယခုလက်ရှိ ငှက်ဖျားရောဂါ တိုက်ဖျက်ရေးတွင် အသုံးပြုနေသော ပြည်သူလူထု အခြေပြု ငှက်ဖျား ကျန်းမာရေးစောင့်ရှောက်မှု ပုံစံ အပေါ် အမြင် နှင့်ရှုထောင့် များကို ဖော်ထုတ်စုစည်းရန်။
- ပြည်သူလူထု အခြေပြု ငှက်ဖျားကျန်းမာရေး စေတ နာ့ဝန်ထမ်းများ၏ ငှက်ဖျားရောဂါ တိုက်ဖျက်နှိမ်နင်းရေး လုပ်ငန်းများ အပေါ် စိတ်အားထက်သန်မှု နှင့် ၄င်းတို ့၏ လူထု အတွင်း အရေးပါမှုကို ထိန်းသိမ်း နိုင်မည့်နည်းလမ်းများကို ဖော်ထုတ်စုစည်းရန်။
- ငှက်ဖျားရောဂါ ထိမ်းချုပ်ရေးမှ ဒေသ တွင်း ငှက်ဖျားရောဂါကင်းဝေးပပျောက်ရေးသို့ အသွင်ကူးပြောင်းရာတွင် ပြည်သူလူထု အခြေပြု ငှက်ဖျားကျန်းမာရေးစောင့်ရှောက်မှု ပုံစံ နှင့် သက်ဆိုင်သော သတိထား ဂရုပြုရမည့် အချက်အလက်များကို ဖော်ထုတ်ရန်။

ကျွန်ုပ်တို့သည် သင်အပါအဝင် ကျန်းမာရေးနှင့် အားကစားဝန်ကြီးဌာနမှ ဝန်ထမ်းများ၊ ငှက်ဖျားဆိုင်ရာ လုပ်ငန်းများ ဆောင်ရွက်နေသည့် အစိုးရမဟုတ်သောအဖွဲ့ အစည်းများမှ ဝန်ထမ်းများ၊ လူထု အတွင်း ဦးဆောင်ဦးရွက်ပြုနေသူများနှင့် လူထုထံမှ သတင်း အချက်အလက်များရယူမည်ဖြစ်ပါသည်။

သုတေသနဆောင်ရွက်ပုံအမျိုးအစား

ဤသုတေသနတွင် သင့်အား သုတေသန၏ တစ်စိတ်တပိုင်းဖြစ်သော ပူးပေါင်းပါဝင်အလုပ်ရုံဆွေးနွေးပွဲတွင် ပါဝင်ရန်ဖိတ်ခေါ် ပါသည်။ ဤဆွေးနွေးခြင်းတွင် သင်၏ရပ်ရွာအတွင်း လက်ရှိငှက်ဖျားရောဂါ အခြေအနေနှင့် ဦးစားပေး ပြည်သူ့ ကျန်းမာရေးပြဿနာများ၊ ငှက်ဖျားထိန်းချုပ်ရေးဆောင်ရွက်ချက်များ နှင့်သင်၏ လူ့ အဖွဲ့ အစည်းတွင်ရရှိနိုင်သောကျန်းမာရေးစောင့်ရှောက်မှုလုပ်ငန်းများ၊ လက်ရှိ ပြည်သူလူထု အခြေပြု ငှက်ဖျား ကျန်းမာရေးစောင့်ရှောက်မှု ပုံစံ အပေါ် အမြင် နှင့်ရှုထောင့် များ၊ မြန်မာနိုင်ငံ ငှက်ဖျားရောဂါ အမြစ်ပြတ်ရှင်းလင်းရေး အတွက် မူဝါဒ နှင့် မဟာဗျူဟာ အားသာချက်နှင့် အားနည်းချက်များ၊ ငှက်ဖျားရောဂါ ထိန်းချုပ်ရေးနှင့်အမြစ်ပြတ်ရှင်းလင်းရေး အတွက်သင်၏ပတ်ဝန်းကျင်တွင်ရရှိနိုင်သောကူညီထောက်ပံ့မှုများ၊ ပြည်သူလူထု အခြေပြု ငှက်ဖျားကျန်းမာရေး စေတ နာ့ဝန်ထမ်းများ၏ ငှက်ဖျားရောဂါ တိုက်ဖျက်နှိမ်နင်းရေး လုပ်ငန်းများ အပေါ် စိတ်အားထက်သန်မှု နှင့် ၄င်းတို့၏လူထုအတွင်းအရေးပါမှုကိုထိန်းသိမ်းနိုင်မည့်နည်းလမ်းများ၊ နှင့် ဓလေ့ထုံးစံ၊လူမှုကျင့်ဝတ်ပိုင်းဆိုင်ရာလက်ခံယုံကြည်မှုတို့ ကြောင့်ငှက်ဖျားရောဂါထိန်းချုပ်ရေးနှင့် အမြစ်ပြတ်ရှင်းလင်းရေးလုပ်ငန်းစဉ်များတွင် ဖြစ်ပေါ် လာနိုင်သော အတားအဆီးနှင့် အားသာချက်များစသည့် အကြောင်းအရာများကို အတူတကွဆွေးနွေးအဖြေရှာပါမည်။

သုတေသနတွင် ပါဝင်မည့်သူများကို ရွေးချယ်ခြင်း

သင့်အား ဤသုတေသနတွင် ပါဝင်ရန်ရွေးချယ်ရခြင်းမှာအရပ်ဖက်ခေါင်းဆောင်တစ်ဦးအနေဖြင့် သင်၏ အတွေ့အကြုံနှင့် အသိပညာများသည် ဤသုတေသနလုပ်ငန်းအတွက်အလွန်အထောက်အကူပေးနိုင်မည်ဟုယုံကြည်သောကြောင့်ဖြစ်ပါသည်။

မိမိဆန္ဒအလျောက် သုတေသနတွင်ပါဝင်ခြင်း

သင်၏ ပါဝင်မှုသည် သင်၏ လွတ်လပ်သော ဆန္ဒအလျောက် ဖြစ်ပါသည်။ အကယ်၍ ပါဝင်ရန် ဆန္ဒမရှိပါလျှင်လည်း သင်၏ လုပ်ငန်းများ နှင့် ကျန်းမာရေးစောင့်ရှောက်မှု အပေါ် သက်ရောက်မှုတစ်စုံတစ်ရာ ရှိမည်မဟုတ်ပါ။ ဤသုတေသနလုပ်ငန်းမှ အချိန်မရွေး အကြောင်းပြချက် မလိုဘဲ နှုတ်ထွက်ခွင့်ရှိပြီး ဤသို့နှုတ်ထွက်ပါကလည်း သင်၏ လုပ်ငန်းများ နှင့် ကျန်းမာရေးစောင့်ရှောက်မှုအား မည်သို့မျှထိခိုက်စေမည် မဟုတ်ပါ။

သုတေသနလုပ်ငန်းလုပ်ဆောင်ချက်အဆင့်ဆင့်

ဆွေးနွေးပွဲကိုအဓိကသုတေသီဒေါက်တာဂင်းဟန်ဦးနှင့် သုတေသနအဖွဲ့ မှ ပြည်တွင်းအဖွဲ့ ဝင်အနည်းဆုံးနှစ်ဦးမှ ဦးဆောင်ဆွေးနွေးပါမည်။ ဆွေးနွေးပွဲတွင်ပါဝင်သူများသည် လူမှုအသိုက်အဝန်း (သို့) ကျေးရွာအသီးသီး ကို ကိုယ်စားပြုသော သင်ကဲ့သို့ အရပ်ဖက်ခေါင်းဆောင်များဖြစ်ကြသည်။ သင်၏သဘောတူညီမှုရရှိပြီးသောအခါ ဆွေးနွေးပွဲရှိဆွေးနွေးချက်များကို အသံသွင်းယူခြင်းဖြင့်၎င်း၊ ရေး၍ ခစ်မှတ်သားခြင်းဖြင့်၎င်း မှတ်တမ်းယူ၍သိမ်းဆည်းထားမည်ဖြစ်သည်။ ဆွေးနွေးခြင်းကို ဘုရားရှိခိုးကျောင်း၊ အဖွဲ့ အစည်းခေါင်းဆောင်၏အိမ် ကဲ့သို့ သော လုံခြုံမှုရှိပြီး အခြားသူများမကြားနိုင်သော နေရာတွင်ပြုလုပ်ပါမည်။

ဆွေးနွေးပွဲတွင် ပါဝင်မည့်ခေါင်းစဉ်များမှာ- (၁) ငှက်ဖျားရောဂါအကြောင်းမိတ်ဆက်ခြင်း နှင့် ဆွေးနွေးပွဲတွင်ပါဝင်ကြသူများ၏ကျေးရွာများရှိ လက်ရှိငှက်ဖျားရောဂါအခြေအနေ၊ (၂) လက်ရှိ ငှက်ဖျားရောဂါတိုက်ဖျက်ရေးအတွက် လုပ်ဆောင်နေမှုများ၊ (၃)ရပ်ရွာအတွင်းရှိ ငှက်ဖျားရောဂါအတွက် ဝန်ဆောင်မှု ပေးနေမှု ပုံစံ၊ (၄) ငှက်ဖျားကောဂါထိန်းချုပ်ရေးမှ အမြစ်ဖြတ်ရှင်းလင်းရေး ကူးပြောင်းမှု အခြေအနေနှင့် လူထုအပေါ် အကျိုးသက်ရောက်မှုများ၊ (၅) စဉ်းစားတွေးခေါ် အဖြေထုတ်ရန်များ(၆) အကြောင်းအရာတစ်ခုခြင်းစီအတွက် အုပ်စုဖွဲ့ ့ဆွေးနွေးခြင်းများ(၇)ဆွေးနွေးပွဲမှ နိဂုံးချုပ် သုံးသပ်ချက်နှင့် အုပ်စုများ၏ အကြံပေးဆွေးနွေးချက်များတို့ ဖြစ်သည်။ စဉ်းစားတွေးခေါ် အဖြေထုတ်သောကဏ္ဍအတွက်သင်၏အတွေ့အကြုံနောက်ခံပေါ် မူတည်ပြီးအုပ်စုဖွဲ့ဆွေးနွေးသွားမည်ဖြစ်ပြီး ပူးပေါင်းပါဝင်သင်ကြားလက်တွေ့ လုပ်ဆောင်နိုင်သော သင်ထောက်ကူကိရိယာများကို အသုံးပြုသွားမည်ဖြစ်သည်။ ဆွေးနွေးပွဲမစမီတွင် လူမှု ့အဖွဲ့ အတွင်းရှိ မိမိ၏ တာဝန်ယူတာဝန်ခံမှု ့အပိုင်း၊ အသက်၊ နေထိုင်သောမြို့ နယ် အစရှိသည့်တစ်ဦး တစ်ယောက်ကို ဖော်ထုတ်သတ်မှတ်နိုင်ခြင်းမပြုသည့် အချက်အလက်များကို ဦးဆောင်ဆွေးနွေးသူမှမေးမြန်း ရယူမည်ဖြစ်သည်။

အချိန်ကြာမြင့်မှု

ဆွေးနွေးပွဲ၏ ကြာမြင့်ချိန်မှ် တစ်ရက်ဖြစ်ပါသည်။(မနက် ၉နာရီ- ညနေ ၅နာရီ)

အချက်အလက်များကို လျှို့ ဝှက်ထားရှိမှု

ဤသုတေသနမှ ရရှိသော သတင်းအချက်အလက်များကို လျှို့ ဝှက်ထားရှိပါမည်။ သုတေသနအဖွဲ့ ဝင်မှလွဲ၍ မည်သူတစ်ဦးတစ်ယောက်မျှ ကြည့်ရှုနားဆင်ပိုင်ခွင့် မရှိပါ။ ဤသုတေသနကို ဆောင်ရွက်သူများသာလျှင် သင်ပြောပြသော အချက်အလက်များကို ရရှိနိုင်မည် ဖြစ်သည်။ သင်၏ အမည် (သို့မဟုတ်) အခြား သင့်ကို မှတ်သားဖေါ်ထုတ်နိုင်သော

အချက်အလက်များကို မှတ်သားဖေါ်ပြထားမည် မဟုတ်ပါ။ သင်ပါဝင်ရန် သဘောတူပါက သင့်အတွက် အမည်လွှဲတစ်ခုဖြင့် သတ်မှတ်ထားပြီး သုတေသနလုပ်ငန်းစဉ်တစ်လျှောက် သင်၏ လွတ်လပ်မှုကို ကာကွယ်ပေးထားမည်ဖြစ်သည်။

ဤမှတ်စုများ နှင့် အသံသွင်းထားသော ကိရိယာများ အားလုံးကို ဘီရိုထဲတွင် သော့ခတ်ပြီးသိမ်းဆည်းထားမည်ဖြစ်၍ သုတေသနဆောင်ရွက်သူများကသာ ရယူနိုင်ပါမည်။ ထို့အတူ ဤသဘောတူခွင့်ပြုလွှာတွင် လက်မှတ်ရေးထိုးခြင်းဖြင့် The Alfred Office of Ethics & Research Governance ၏ ဆန်းစစ်မှု ဘုတ်အဖွဲ့နှင့် ဆေးသုတေသနဦးစီးဌာန လူပုဂ္ဂိုလ်များအပေါ် သုတေသန စမ်းသပ်မှုဆိုင်ရာ ကျင့်ဝတ်ကော်မီတီတို့၏ ကိုယ်စားလှယ်များကလေ့လာမှု၏ မှတ်တမ်းများကို ကြည့်ရှုနိုင်ရန် သဘောတူခွင့်ပြုချက် ပေးခြင်းဖြစ်သည်။ သို့သော် ထိုအဖွဲ့များက သင်၏ ကိုယ်ပိုင်လွတ်လပ်ခွင့်ကို ထိန်းသိမ်းကာကွယ်ပေးထားကြောင်း သေချာစေရန်အလို့ငှာသာ ကြည့်ရှု စစ်ဆေးမည်ဖြစ်ပါသည်။ အသံ မှတ်တမ်း များကို နားထောင်ပြီး ဆွေးနွေးပြောဆိုမှုများကို ရေးသားမှတ်တမ်းတင်မည် ဖြစ်ပါသည်။ အသုံးပြုထားသော အသံသွင်းစက်အတွင်းရှိ အသံ မှတ်တမ်း များကို လျှို့ဝှက် ဂဏန်းဖြင့် ကာကွယ်ထားသော ကွန်ပျူတာထဲသို့ ကူးယူပြီးနောက် ဖျက်ဆီးပစ်မည်ဖြစ်ပါသည်။ ရေးသားထားသော မှတ်တမ်းများနှင့် အခြားမှတ်သားချက်များ အားလုံးအား ၇ နှစ် အထိ လုံခြုံစွာ သိမ်းဆည်းထားမည် ဖြစ်ပြီး၊ ၇ နှစ်ကျော်ပါက ဖျက်ဆီး ပစ်မည်ဖြစ်ပါသည်။

ထိခိုက်နိုင်မှုနှင့် ကိုယ်စိတ် အနှောင့်အယှက်ဖြစ်စေခြင်းများ

ဤဆွေးနွေးခြင်းတွင် သင်၏ရပ်ရွာအတွင်း လက်ရှိငှက်ဖျားရောဂါ အခြေအနေနှင့် ဦးစားပေး ပြည်သူ့ ကျန်းမာရေးပြဿနာများ၊ ငှက်ဖျားထိန်းချုပ်ရေးဆောင်ရွက်ချက်များ နှင့်သင်၏ လူ့ အဖွဲ့ အစည်းတွင်ရရှိနိုင်သောကျန်းမာရေးစောင့်ရှောက်မှုလုပ်ငန်းများ၊ လက်ရှိ ပြည်သူလူထု အခြေပြု ငှက်ဖျား ကျန်းမာရေးစောင့်ရှောက်မှု ပုံစံ အပေါ် အမြင် နှင့်ရှုထောင့် များ၊ မြန်မာနိုင်ငံ ငှက်ဖျားရောဂါ အမြစ်ပြတ်ရှင်းလင်းရေး အတွက် မူဝါဒ နှင့် မဟာဗျူဟာ အားသာချက်နှင့် အားနည်းချက်များ၊ ငှက်ဖျားရောဂါ ထိန်းချုပ်ရေးနှင့်အမြစ်ပြတ်ရှင်းလင်းရေး အတွက်သင်၏ပတ်ဝန်းကျင်တွင်ရရှိနိုင်သောကူညီထောက်ပံ့မှုများ၊ ပြည်သူလူထု အခြေပြု ငှက်ဖျားကျန်းမာရေး စေတနာ့ဝန်ထမ်းများ၏ ငှက်ဖျားရောဂါ တိုက်ဖျက်နှိမ်နင်းရေး လုပ်ငန်းများ အပေါ် စိတ်အားထက်သန်မှု နှင့် ၎င်းတို့၏လူထုအတွင်းအရေးပါမှုကိုထိန်းသိမ်းနိုင်မည့်နည်းလမ်းများ၊ နှင့် ဓလေ့ထုံးစံ၊လူမှုကျင့်ဝတ်ပိုင်းဆိုင်ရာလက်ခံယုံကြည်မှုတို့ ကြောင့်ငှက်ဖျားရောဂါထိန်းချုပ်ရေးနှင့် အမြစ်ပြတ်ရှင်းလင်းရေးလုပ်ငန်းစဉ်များတွင် ဖြစ်ပေါ်လာနိုင်သော အတားအဆီးနှင့် အားသာချက်များစသည့် အကြောင်းအရာများကို ဝင်ရောက်ဆွေးနွေးရန်ဖိတ်ခေါ်ပါမည်။

မေးခွန်းများကိုဖြေကြားရာနှင့် ပူးပေါင်းပါဝင်သင်ကြားလက်တွေ့ လုပ်ဆောင်နိုင်သောသင်ထောက်ကူကိရိယာများဖြင့် ဆွေးနွေးရာတွင် အနည်းငယ် စိတ်မသက်မသာဖြစ်နိုင်ပါသည်။ သို့ရာတွင် သင့်အား စိတ်အနှောင့် အယှက်ဖြစ်စေမည့် အကြောင်းအရာများ ပါဝင်မှုမရှိစေရန် မျှော်လင့်ပါသည်။ အချို့သောအကြောင်းအရာများ၊သင်ကြားလုပ်ဆောင်မှုများ သည် သင့်အား စိတ်အနှောင့် အယှက်ဖြစ်စေမည်ဆိုလျှင် မဖြေဘဲ(သို့) ဝင်ရောက်ပါဝင်ခြင်းမပြုဘဲ ထားနိုင်ပါသည်။

အကျိုးကျေးဇူးများ

ဤသုတေသနတွင်ပါဝင်၍ သင့်အတွက် တိုက်ရိုက်အကျိုးကျေးဇူးမရှိပါ။ သို့သော် သင်ပါဝင် ဖြေကြားပေးသည့်အတွက် အဓိက သုတေသီ ဒေါက်တာဝင်းဟန်ဦး ၏ ပြည်သူလူထု အခြေပြု ငှက်ဖျား ကျန်းမာရေးစောင့်ရှောက်မှု ပုံစံ အား သုတေသနပြုတည်ဆောက်ရာတွင် ကူညီပေးမည် ဖြစ်ပါသည်။

ကျေးဇူးတုံ့ပြန်မှု

သုတေသနတွင် ပါဝင်မှုအတွက် အစားအသောက်၊ သင်၏အချိန်ပေးရမှုအတွက် မြန်မာကျပ်ငွေ ၁၀၀၀၀ ကျပ်တိတိ နှင့် အမှန်တကယ်ကုန်ကျသော ခရီးစားရိတ်တို့ကို ပြန်လည်ထောက်ပံ့မည်ဖြစ်ပါသည်။

သုတေသန၏ ရလဒ်များကို ဖြန့်ဝေခြင်း

ဤသုတေသနရလဒ်များကို ပြည်တွင်းနှင့် ပြည်ပတွင် သုတေသနစာတမ်းအနေဖြင့် ဖြန့်ဝေရန် မျှော်လင့်ပါသည်။ သို့သော်လည်း တစ်ဦးတစ်ယောက်ကို ဖော်ထုတ်သတ်မှတ်နိုင်သည့်သတင်းအချက်အလက်ပါဝင်မည်မဟုတ်ပါ။ သုတေသနရလဒ်များကို အဓိကသုတေသီထံတွင် ၂၀၁၈ခုနှစ် ဧပြီလနောက်ပိုင်းတွင် တောင်းယူနိုင်ပါသည်။

ဆက်လက်မပါဝင်လိုကြောင်း ငြင်းဆန်နိုင်ခွင့်

သင့်အနေနှင့် သုတေသနတွင် ဆက်လက်မပါဝင်လိုတော့လျှင် အချိန်မရွေး ငြင်းဆန်ပိုင်ခွင့်ရှိပြီး ထိုငြင်းဆန်မှုသည် သင်၏ မူလအခွင့်အရေးများကို ထိခိုက်စေမည် မဟုတ်ပါ။

ဆက်သွယ်နိုင်မည့်ပုဂ္ဂိုလ်များ

သုတေသနတွင်ပါဝင်ဆောင်ရွက်ရန်သဘောတူပါက အောက်ပါပုဂ္ဂိုလ်ထံ အချိန်မရွေးဆက်သွယ်မေးမြန်းနိုင်ပါသည်။

ဒေါက်တာ ဝင်းဟန်ဦး

ပါရဂူ ကျောင်းသား

ကျန်းမာရေးဆိုင်ရာ ဌာန၊ Deakin တက္ကသိုလ်

၂၂၆၊ ၄ လွှာ၊ ဦးဝိစာရလမ်း၊ ဝိဇယပလာဇာ၊ ဗဟန်းမြို့နယ်၊ ရန်ကုန်မြို့။

ဖုန်း +95-1-375785, 375763, 375727, 512693 Ext 106

Email: owinhan@deakin.edu.au

ပါဝင်ဆောင်ရွက်သူများ၏ အခွင့်အရေးများနှင့် ပတ်သက်၍ မေးစရာများရှိပါက အောက်ပါ ပုဂ္ဂိုလ်များထံ ဆက်သွယ်နိုင်ပါသည်။

အတွင်းရေးမှူး

လူပုဂ္ဂိုလ်များအပေါ် သုတေသနစမ်းသပ်မှုဆိုင်ရာ ကျင့်ဝတ်ကော်မတီ

ဆေးသုတေသနဦးစီးဌာန၊

အမှတ် ၅ ဇီဝကလမ်း၊ ဒဂုံစာတိုက်၊ ၁၁၁၉၁၊ ရန်ကုန်၊ မြန်မာနိုင်ငံ၊

ဖုန်း ၀၁ ၃၇၅၄၄၇ လိုင်းခွဲ ၁၁၈ (ရုံးချိန်အတွင်း)

Complaints Officer

Office of Ethics & Research Governance, Alfred Health

Phone: +61 3 9076 3619, Email: research@alfred.org.au

Note: You will need to quote the following Alfred Health project number: 445/17

အပိုင်း၂၊ သဘောတူညီချက်

ကျွန်ုပ်သည် "မြန်မာနိုင်ငံ ငှက်ဖျားရောဂါ အမြ စ်ပြတ်ရှင်းလင်းရေး တွင် **ပြည်သူလူထု အခြေပြု ငှက်ဖျား ကျန်းမာရေးစောင့်ရှောက်မှု ပုံစံများ အပေါ် အမြင်နှင့် ရှုထောင့်များကို ဖော်ထုတ်ခြင်း"** လုပ်ငန်းတွင် ပါဝင်ရန် ဖိတ်ခေါ်ခြင်းခံရပါသည်။ သုတေသနပြုလုပ်သော လုပ်ငန်းစဉ်တွင် အချိန် ၈နာရီ ခန့်ကြာမြင့်သော အလုပ်ရုံဆွေးနွေးပွဲပါဝင်ကြောင်းသိရှိနားလည်ပြီး ဖြစ်ပါသည်။က ျွန်ုပ်အတွက် ကိုယ်ရေးကိုယ်တာ အကျိုးအမြတ် ရရှိမည် မဟုတ်ကြောင်းကိုလည်း သိရှိပြီးဖြစ်ပါသည်။ သုတေသနဆောင်ရွက်သူနှင့် ဆက်သွယ်ရန် လိပ်စာနှင့် ဖုန်းနံပါတ်များကိုလည်း သိရှိပြီးဖြစ်ပါသည်။ ကျွန်ုပ်သည် ရှေ့မှအချက်အလက်များကို ဖတ်ရှုပြီးဖြစ်ပါသည် (သို့မဟုတ်) ကျွန်ုပ်အား ဖတ်ပြပြီးဖြစ်ပါသည်။ ကျွန်ုပ်တွင် မေးခွန်းမေးပိုင်ခွင့်နှင့် ထိုမေးခွန်းများကို ကျွန်ုပ်ကျေနပ်သည်အထိ ဖြေကြားပြီး ဖြစ်ပါသ ည်။ ကျွန်ုပ်သည် သုတေသနတွင် မိမိဆန္ဒ အလျောက်ပါဝင်ရန် သဘောတူပါသည်။ ဤသုတေသနလုပ်ငန်းမှ အချိန်မရွေး နုတ်ထွက်ခွင့်ရှိပြီး၊ ယင်းသို့ နုတ်ထွက်ခြင်းကြောင့် ကျွန်ုပ်၏ လုပ်ငန်းများ နှင့် ရပိုင်ခွင့်များကို ထိခိုက်မှုမရှိကြောင်း နားလည်ပြီးဖြစ်သည်။

ပါဝင်သူအမည် ------------------------

ပါဝင်သူလက်မှတ် ---------------------

ရက်စွဲ၊ ---------------------------------

ရက် လ နှစ်

ပါဝင်သူသည် စာမတတ်ပါက စာတတ်သော သက်သေတစ်ဦးမှ လက်မှတ်ရေးထိုးရမည် (ဖြစ်နိုင်ပါက ထိုသက်သေကို ပါဝင်သူမှရွေးချယ်ရမည်ဖြစ်ပြီး သုတေသန အဖွဲ့နှင့် ဆက်စပ်မှု မရှိရပါ)။ စာမတတ်သောပါဝင်သူသည် အောက်တွင်လက်ဗွေနှိပ်ရမည်ဖြစ်သည်။

လက်ဗွေနှိပ်ရန်

ကျွန်ုပ်သည် မေးမြန်းသူကပါဝင်သူအား သဘောတူခွင့်ပြုလွှာကို တိကျသေချာစွာဖတ်ပြခြင်းကို တွေ့ရှိပြီးဖြစ်ပါသည်။ ပါဝင်သူမှလဲ မေးခွန်းများပြန်လည် မေးမြန်းခွင့်ရှိခဲ့ပါသည်။ ပါဝင်ဖြေဆိုသူသည် လွတ်လပ်စွာ မိမိသဘောဆန္ဒအလျောက် ဖြေဆို ခြင်းဖြစ်ကြောင်း အတည်ပြုပါသည်။

သက်သေအမည် ------------------------

သက်သေ၏လက်မှတ် ----------------------

ရက်စွဲ၊ ---------------------------------

ရက် လ နှစ်

ကျွန်ုပ်သည် ပါဝင်ဖြေဆိုရန် အလားအလာရှိသူအား သဘောတူခွင့်ပြုလွှာကို သေချာစွာဖတ်ပြခဲ့ပါသည်။ (သို့မဟုတ်) ဖတ်ပြသည်ကို တွေ့ရှိပါသည်။ ပြီးနောက် မေးခွန်းများ ပြန်လှန်မေးမြန်းခွင့် ပေးခဲ့ပါသည်။ ပါဝင်ဖြေဆိုသူသည် လွတ်လပ်စွာ မိမိသဘောဆန္ဒအလျောက် ဖြေဆို ခြင်းဖြစ်ကြောင်း အတည်ပြုပါသည်။

တွေ့ဆုံမေးမြန်းသူအမည် --------------------------

တွေ့ဆုံမေးမြန်းသူ လက်မှတ် ----------------------

ရက်စွဲ၊ --

ရက် လ နှစ်

ဤသဘောတူခွင့်ပြုလွှာ မိတ္တူတစ်စောင်ကို သုတေသနတွင် ပါဝင်မည့်သူအား ပေးအပ်ပြီး ဖြစ်ပါသည်။--------------------------- (သုတေသီ၏ လက်မှတ်တို)
