# Supplementary material for: Community demand for comprehensive primary health care from malaria volunteers in South-East Myanmar: a qualitative study
Source: Malar J. 2021 Jan 6;20:19. doi: 10.1186/s12936-020-03555-4 (PMC7789746; doi:10.1186/s12936-020-03555-4)
Supplement: Supplementary file 4 — Additional file 4. Approval letters from Myanmar Ministry of Health and Sports and the Data Tranfer Agreement. [file 12936_2020_3555_MOESM4_ESM.pdf]

THE REPUBLIC OF THE UNION OF MYANMAR
MINISTRY OF HEALTH AND SPORTS
DEPATMENT OF PUBLIC HEALTH

Letter No: Pakaya/yana-ngapha/2017/280
Date ..24....7......2017.......

To

Chairman
Ethics Review Committee
Department of Medical Research
Ministry of Health and Sports
The Republic of the Union of Myanmar

Subject: Agreement letter for the Dr Win Han Oo PhD research supported by Burnet Institute.

Dr Win Han Oo submitted a request letter (reference number 061/BIMM/MOHS-Request) dated 18th July 2017 and the research protocol to conduct his PhD research in Myanmar. With reference to the letter and the protocol, we were informed about the aims, objectives and methodologies of the studies named "Perspectives of community delivered models for the malaria elimination in Myanmar: A qualitative study" and "Readiness and acceptability of the new/adapted community delivered model by the community for malaria elimination: A quantitative study".

In order to understand more on the community delivered models and malaria elimination in Myanmar, we, Vector Borne Disease Control Program, have no objection to the proposed studies of Dr Win Han Oo for his PhD supported by Burnet Institute.

Yours sincerely,

Dr Zaw Lin
Deputy Director
Vector Borne Disease Control Program
Department of Public Health
Ministry of Health and Sports

The Government of the Republic of the Union of Myanmar

Ministry of Health and Sports

Department of Public Health

Letter No. PaKaYa / YaNa - 2017/(246)

Date: 10-11-2017

To,

Chairman
Ethics Review Committee
Department of Medical Research
Ministry of Health and Sports
The Republic of the Union of Myanmar

Subject: **Approval for the study entitled "Community Delivered Model(s) for Malaria Elimination in Myanmar [Stage 1: Perspectives of community delivered models for the malaria elimination in Myanmar: A qualitative study and Stage 2: Readiness and acceptability of the new/adapted community delivered model by the community for malaria elimination: A quantitative study]"**

Dear Chairman,

This letter is to verify that Disease Control Division approved the study entitled "**Community Delivered Model(s) for Malaria Elimination in Myanmar [Stage 1: Perspectives of community delivered models for the malaria elimination in Myanmar: A qualitative study and Stage 2: Readiness and acceptability of the new/adapted community delivered model by the community for malaria elimination: A quantitative study]**" for the ethical clearance. The study will be conducted in collaboration with National Malaria Control Programme and Burnet Institute Myanmar.

The Principal Investigator, Dr. Win Han Oo from the Burnet Institute Myanmar has signed the **Data transfer/use agreement** with the national programme for dissemination and publication of the study results. Principal Investigator should put up the protocol for the approval of Ministry of Health and Sports after the ethical clearance.

Yours sincerely,

10/11/2017

(Dr. Thandar Lwin)

Director (Disease Control), Department of Public Health

Cc:

- Deputy Director, National Malaria Control Programme, Department of Public Health
- Office copy

Data Transfer/Use Agreement

This Data Transfer/Use Agreement (DTA) is used to transfer program and clinical data from the National Malaria Control Program (NMCP) to the principal investigator of the research (Recipient).

DTA Reference number:

This Data Transfer Agreement is made by and between the NMCP, Myanmar **(Provider)** and Dr Win Han Oo **(Recipient)** For the purpose of the research project **"Community Delivered Model(s) for Malaria Elimination in Myanmar [Stage 1: Perspectives of community delivered models for the malaria elimination in Myanmar: A qualitative study and Stage 2: Readiness and acceptability of the new/adapted community delivered model by the community for malaria elimination: A quantitative study]"** and hereby agree to the following terms and conditions;

1. The data to be provided to recipient which are listed under Annex 1 – Data to be transferred / used.
2. The data shall be used exclusively for non-commercial, non-military scientific research by the recipient. The data shall be used only at the recipient organization under the direct responsibility of the recipient scientist.
3. The data shall be used in compliance with all applicable statutes, regulations and ethical requirements.

Confidentiality and Protection

4. The recipient scientist/s hereby confirm that they shall adhere to information technology best practices in all aspects of management and use of the data, and shall provide appropriate safeguards and controls to ensure the security of the data and protection of data confidentiality at all times. In particular, the recipient shall keep the data and related confidential information in a secure environment, protected against theft, damage, loss, misuse or unauthorized access.
5. Under no circumstances shall the recipient scientist and the recipient's authorized personnel attempt to identify specific individuals from any of the Data received.
6. The data shall be treated as confidential at all times as well as any and all information received in relation to the data. The recipient shall not disclose it to any third parties without the prior written agreement of provider.

Ownership

7. The ownership of the data shall remain with the provider.
8. Recipient shall use the data solely for the research study stated in Annex 2.

Ethical Adherence

9. All research projects using the data shall obtain approval from Ethics Review Committee (Department of Medical Research, Ministry of Health and Sports, Myanmar) and Alfred Ethic Committee (Alfred Hospital, Melbourne, VIC, Australia). It is the responsibility of recipient to seek for approval of the study from the relevant authority.

Publications

10. Recipient shall consult with the provider prior to the preparation and submission of presentation/publication materials, which involve the data.

11. Recipient agrees to provide the provider with a copy of any publication, which contains results obtained from the use of the data. The recipient shall recognize the provider as co-author in all publications containing any data or information about the data unless the provider indicates otherwise.

Liabilities

12. The provider agree to use the data by the recipient in accordance with the terms explicitly provide in this agreement.

Amendment and Termination

13. This Agreement will terminate on the earliest of the following dates:

a. Three (3) years after completion of the current research with the data, or
b. On thirty (30) days written notice by the provider;

If termination should occur under 13(a) or (b) above, the recipient, will discontinue its use of the data and will, upon direction of the provider, return or destroy the data.

This Agreement is duly signed by:

PROVIDER INSTITUTION/SCIENTIST	RECIPIENT SCIENTIST
Signature	Signature
Date: 9.11.2017	Date: 8.11.17
Printed Name: Dr Aung Thi	Printed Name: Dr Win Han Oo
Designation: Programme Manager	Designation: PhD Candidate
National Malaria Control Programme	(Deakin University)
Department of Public Health	Burnet Institute
Ministry of Health and Sports	Melbourne, Australia
Myanmar	

ANNEX 1. Data to be transferred / used

[Detail description of Requested data and data variable should be listed here]

**Stages**	**Variables**	**Data source**
Stage 1- interviews	- Current malaria situation and control measures<br>- Views and perspectives on the current malaria CHW model<br>- Policy and strategic barriers and enablers for Myanmar malaria elimination<br>- Operational barriers and enablers for Myanmar malaria control and elimination using community delivered models<br>- Strategies to maintain the motivation and social role of CHWs in the community<br>- The factors need to be addressed during the transition from malaria control to elimination using community delivered models<br>- Additional topic identified by the interviewee	MoHS and malaria implementing partner stakeholders interviews (audio-recordings and field notes)
Stage 1 – workshops	- Current malaria situation and priority health problems in the respective community<br>- Malaria control measures and available health services in the respective community<br>- Views and perspectives on the current malaria CHW models<br>- Policy and strategic barriers and enablers for effective malaria control and elimination in their community<br>- Available community supports for malaria control and elimination in their community<br>- Strategies to maintain the motivation and social role of CHWs in the community<br>- Culture, customs and norms of the ethnic communities that play as barriers and enablers for effective malaria control and elimination in their community	Participatory workshops with community leaders (audio-recordings and field notes)

Stage 1 – FGDs	- Current malaria situation and priority health problems in your community<br>- Malaria control measures and available health services in the respective community<br>- Views and perspectives on the current malaria CHW models<br>- Available community supports for malaria control and elimination in their community<br>- Strategies to maintain the motivation and social role of CHWs in the community<br>- Culture, customs and norms of the ethnic communities that play as barriers and enablers for effective malaria control and elimination in their community	Focus Group Discussion (FGD) with community members (audio-recordings and field notes)
Stage 2 - Survey	- Demographic information of the community<br>- Readiness and acceptability of the community for malaria diagnosis, treatment and referral, prevention and reporting, other health services, and overall acceptability and readiness for new model.	Community survey form

ANNEX 2. Description of Research Project

Title: Community Delivered Model(s) for Malaria Elimination in Myanmar
[Stage 1: Perspectives of community delivered models for the malaria elimination in Myanmar: A qualitative study and Stage 2: Readiness and acceptability of the new/adapted community delivered model by the community for malaria elimination: A quantitative study]

Principal investigator: Win Han Oo, PhD Candidate (Deakin University), Burnet Institute, Melbourne, Australia, 099 74902209, winhan.oo@burnet.edu.au

Co-Investigators: Freya Fowkes[1], Lisa Gold[2], Elizabeth Hoban[2], Paul Agius[1], Kyu Kyu Than[1], Zaw Lin[3], Aung Thi[3], Thazin La[4]

Associate Investigators: Naanki Pasricha[1], Phone Myint Win[4], Aung Ko Ko[4], Khine Soe Lin[4], Win Lei Yee[4]

[1] Burnet Institute, Melbourne, VIC, Australia
[2] School of Health and Social Development, Faculty of Health, Deakin University, Melbourne, VIC, Australia
[3] Vector Borne Disease Control Programme, Department of Public Health, Ministry of Health and Sports, Myanmar
[4] Burnet Institute Myanmar, Yangon, Myanmar

Introduction: Myanmar has the highest burden of malaria in the Greater Mekong Sub-region and aims to eliminate malaria by 2030. Over the past decade, the National Malaria Control Program (NMCP) and malaria implementing partners have used malaria Community Health Workers (CHW) to deliver effective and cost-effective malaria control to reduce the burden of malaria. However, along with the successful reduction of malaria cases in endemic areas, the interest in seeking out malaria services from CHW, as well as the community trust in them, has declined. In the current elimination phase, when malaria prevalence is very low, the community delivered CHW model needs to be adapted according to the changing context, to maintain the effectiveness and cost effectiveness of malaria control and elimination measures.

Aim: This PhD project aims to develop a community-delivered malaria elimination model that is acceptable, operational, pragmatic, evidence based and effective compared to the traditional model for malaria elimination in Myanmar.

Method – stage 1: A qualitative research study named "Perspectives of community delivered models for the malaria elimination in Myanmar: A qualitative study" will be performed to

explore the perspectives, views and inputs of key health stakeholders and community members in Myanmar on community-delivered models for malaria elimination in Myanmar. This qualitative research will apply Focus Group Discussions, participatory workshops, In-depth Interviews and Key Informant Interviews. A range of broad population groups will be targeted for this study: Myanmar Ministry of Health and Sports (MoHS) stakeholders, malaria Implementing Partner (IP) stakeholders, community leaders and community members ranging from the national stakeholders level to the grass roots level community members and decision makers. Results from the qualitative research study will ultimately contribute to the construction of the adapted community-delivered model.

Method – stage 2: After the model construction, the readiness and acceptability of the model by the community members will be assessed using a quantitative survey named "Readiness and acceptability of the new/adapted community delivered model by the community for malaria elimination: A quantitative study". 300 community members of villages in South East Myanmar (Kayin and Kayah States) will be recruited to take part in the survey. The main analysis will focus on how community members feel about individual components of the new model and whether they accept it.

Ethical considerations: Written informed consent will be obtained from all participants and the data will be handled with confidential and ethical standards for both studies. No ethical issue apart from the potential discomfort of participants during the data collection has been identified.

Dissemination of the findings: The findings will be incorporated into the PhD thesis of PI Win Han Oo and will be submitted to Deakin University. Findings from the study may also be submitted for publication in peer-reviewed journals and abstracts for relevant conference presentations. The studies are supported technically and financially by Deakin University and Burnet Institute.
