# Supplementary material for: Community demand for comprehensive primary health care from malaria volunteers in South-East Myanmar: a qualitative study
Source: Malar J. 2021 Jan 6;20:19. doi: 10.1186/s12936-020-03555-4 (PMC7789746; doi:10.1186/s12936-020-03555-4)
Supplement: Supplementary file 5 — Additional file 5. Ethics Review Committee Certificates of approval. [file 12936_2020_3555_MOESM5_ESM.pdf]

The Government of the Republic of the Union of Myanmar

Ministry of Health and Sports

Department of Medical Research

No. 5, Ziwaka Road, Dagon Township, Yangon 11191

Tel : 95-1-375447, 95-1-375457, 95-1-375459 Fax : 95-1-251514

ERC Number: 012917
Approval Number: Ethics/DMR/2017/150
Date of Approval: 15 December, 2017 (valid up to 14 December, 2018)

Project Title: **Community Delivered Model(s) for Malaria Elimination in Myanmar [Perspectives of community delivered models for the malaria elimination in Myanmar: A qualitative study]**

Principal Investigator: Dr. Win Han Oo
Deakin University, Melbourne, Australia

Documents Accepted:

1. Ethical Proposal Form Version Dated 20 November, 2017
2. Full Proposal Protocol Version Dated 20 November, 2017
3. Proposal Summary Version Dated 20 November, 2017
4. Agreement to comply with ethical guideline 20 November, 2017
5. Qualitative guides for data collection (English & Myanmar) Dated 20 November, 2017
6. Informed Consent forms (English & Myanmar) Version Dated 20 November, 2017
7. Ethics approval letter from The Alfred Ethics Committee Dated 8 September, 2017
8. Approval letter from Department of Public Health Dated 10 November, 2017
9. Data Transfer/Use Agreement letter Dated 9 November, 2017
10. Copy of MOU Dated 25 September, 2017
11. Investigators' CV Dated 20 November, 2017

The Ethics Review Committee on Medical Research Involving Human Subjects, Department of Medical Research, Ministry of Health and Sports approves to conduct the proposed research project as it is in full compliance with the Declaration of Helsinki, Council for International Organizations of Medical Sciences guidelines and International Conference on Harmonisation in Good Clinical Practice guidelines.

Prof. Pe Thet Khin
Chairperson
Ethics Review Committee
Department of Medical Research

The Government of the Republic of the Union of Myanmar
Ministry of Health and Sports
Department of Medical Research
No. 5, Ziwaka Road, Dagon Township, Yangon 11191
Tel : 95-1-375447, 95-1-375457, 95-1-375459 Fax : 95-1-251514

Approval is subject to following conditions:

- The principal investigator (PI) must notify immediately to the ERC of any changes or deviation in the conduct of the research activity. Only with the ERC's approval such changes in the study must be pursued. The PI must also make a prompt report to the ERC of any new and significant information that may impact a research subject's safety or willingness to continue in the study and any anticipated problems involving risks to the participants or other.

- PI is responsible for submitting the progress report at least 6 weeks prior to the expiry of the approved date to allow adequate time for the ERC for substantive and meaningful review and for assuring that the research is not conducted beyond the approved date.

- Final report is to be provided to ERC at the end of the study.

- Random site visits may be carried out to ensure that informed consent procedures are appropriate.

ETHICS COMMITTEE CERTIFICATE OF APPROVAL

This is to certify that

Project No: 445/17

Project Title: Community Delivered Model(s) for Malaria Elimination in Myanmar (“Perspectives of community delivered models for the malaria elimination in Myanmar: A qualitative study” and “Readiness and acceptability of the new/adapted community delivered model by the community for malaria elimination: A quantitative study”)

Principal Researcher: Dr Win Han Oo (Supervisor: Dr Freya Fowkes)

Protocol Version 2 **dated:** 8-Sep-2017

Protocol Appendix A: Qualitative Research Guides Version 1 dated: 23-Aug-2017
Protocol Appendix B: Questionnaire for the survey Version 1 dated: 23-Aug-2017

Protocol Appendix C: Informed Consent Form for a Community Survey Version 1 dated: 23-Aug-2017
Protocol Appendix C: PICF - Semi-structured Interview (MoHS stakeholders) Version 2 dated: 8-Sep-2017
Protocol Appendix C: PICF - Semi-structured interview (malaria implementing partner stakeholders) Version 2 dated: 8-Sep-2017
Protocol Appendix C: PICF - Participatory workshop with community leaders Version 2 dated: 8-Sep-2017
Protocol Appendix C: PICF - Focus Group Discussion (FGD) with community members Version 2 dated: 8-Sep-2017

Protocol Appendix D: Screening tool for the survey Version 2 dated: 8-Sep-2017

Participant Information and Consent Form Version dated:

*was considered by the Ethics Committee on **21-Sep-2017**, meets the requirements of the National Statement on Ethical Conduct in Human Research (2007) and was **APPROVED** on **21-Sep-2017***

It is the Principal Researcher’s responsibility to ensure that all researchers associated with this project are aware of the conditions of approval and which documents have been approved.

The Principal Researcher is required to notify the Secretary of the Ethics Committee, via amendment or progress report, of

- Any significant change to the project and the reason for that change, including an indication of ethical implications (if any);
- Serious adverse effects on participants and the action taken to address those effects;
- Any other unforeseen events or unexpected developments that merit notification;
- The inability of the Principal Researcher to continue in that role, or any other change in research personnel involved in the project;
- Any expiry of the insurance coverage provided with respect to sponsored clinical trials and proof of re-insurance;
- A delay of more than 12 months in the commencement of the project; and,
- Termination or closure of the project.

Additionally, the Principal Researcher is required to submit

- A Progress Report on the anniversary of approval and on completion of the project *(forms to be provided)*;

The Ethics Committee may conduct an audit at any time.

All research subject to the Alfred Hospital Ethics Committee review must be conducted in accordance with the National Statement on Ethical Conduct in Human Research (2007).

The Alfred Hospital Ethics Committee is a properly constituted Human Research Ethics Committee in accordance with the National Statement on Ethical Conduct in Human Research (2007).

SPECIAL CONDITIONS
The project should not commence until all approvals from other ethical review bodies have been granted, and copies of those approvals and any changes requested by those review bodies have been provided to the Alfred Hospital Ethics Committee.

SIGNED:

Professor John J. McNeil
Chair, Ethics Committee

Please quote project number and title in all correspondence

theAlfred

Ethics Committee

Certificate of Approval of Amendments

This is to certify that amendments to

Project: **445/17 Community Delivered Model(s) for Malaria Elimination in Myanmar (“Perspectives of community delivered models for the malaria elimination in Myanmar: A qualitative study” and “Readiness and acceptability of the new/adapted community delivered model by the community for malaria elimination: A quantitative study”)**

Principal Researcher: **Dr Win Han Oo**

Amendment:

Changes to documents requested by Ethics Review Committee, Department of Medical Research, Ministry of Health & Sports, Republic of the Union of Myanmar
Full Proposal, Protocol and Appendices **Version 3** dated: **20-Nov-2017**

have been approved in accordance with your amendment application dated **19-Dec-2017** on the understanding that you observe the National Statement on Ethical Conduct in Human Research.

It is now your responsibility to ensure that all people associated with this particular research project are made aware of what has actually been approved and any caveats specified in correspondence with the Ethics Committee. Any further change to the application which is likely to have a significant impact on the ethical considerations of this project will require approval from the Ethics Committee.

Professor John J. McNeil
Chair, Ethics Committee

Date: **22-May-2018**

All research subject to Alfred Hospital Ethics Committee review must be conducted in accordance with the National Statement on Ethical Conduct in Human Research (2007).

The Alfred Ethics Committee is a properly constituted Human Research Ethics Committee operating in accordance with the National Statement on Ethical Conduct in Human Research (2007).

Human Research Ethics

Deakin Research Integrity
Burwood Campus Victoria
Postal: 221 Burwood Highway
Burwood Victoria 3125 Australia
Telephone 03 9251 7123
research-ethics@deakin.edu.au

Memorandum

To: A/Prof Lisa Gold
Population Health
B

cc: Dr Win Han Oo

From: Deakin University Human Research Ethics Committee (DUHREC)

Date: 27 October, 2017

Subject: 2017-328

Community Delivered Model(s) for Malaria Elimination in Myanmar
(?Perspectives of community delivered models for the malaria elimination in Myanmar: A

Please quote this project number in all future communications

Approval granted by Alfred Health HREC for this project will be noted at the DUHREC meeting to be held on 13/11/2017.

It will be noted that approval has been granted for A/Prof Lisa Gold, Population Health, to undertake this project as stipulated in Alfred Health HREC approval documentation.

The approval noted by the Deakin University Human Research Ethics Committee is given only for the project and for the period as stated in the memo. It is your responsibility to contact the HREC should the project be discontinued before the expected date of completion. You are reminded that:

The Deakin logo should be on any participant documents, including the Plain Language Statement, or where that is not possible, ensure Deakin University's involvement in the project is clearly written in the documentation
The Deakin Human Research Ethics Office needs to be notified immediately if any complaints are received
An annual/progress report must be submitted to the approving HREC and at the conclusion of the project, a final report must be submitted to the Deakin HREC.

DUHREC may need to audit this project as part of the requirements for monitoring set out in the National Statement on Ethical Conduct in Human Research (2007).

Human Research Ethics Unit
research-ethics@deakin.edu.au
Telephone : 03 9251 7123
